# Cu(I) and Cu(II) Complexes Based on Lonidamine-Conjugated Ligands Designed to Promote Synergistic Antitumor Effects

**DOI:** 10.1021/acs.inorgchem.1c03658

**Published:** 2022-03-14

**Authors:** Fabio Del Bello, Maura Pellei, Luca Bagnarelli, Carlo Santini, Gianfabio Giorgioni, Alessandro Piergentili, Wilma Quaglia, Chiara Battocchio, Giovanna Iucci, Irene Schiesaro, Carlo Meneghini, Iole Venditti, Nitya Ramanan, Michele De Franco, Paolo Sgarbossa, Cristina Marzano, Valentina Gandin

**Affiliations:** † School of Pharmacy, Medicinal Chemistry Unit, University of Camerino, via S. Agostino 1, 62032 Camerino, Italy; ‡ School of Science and Technology, Chemistry Division, University of Camerino, via S. Agostino 1, 62032 Camerino, Italy; § Department of Science, Roma Tre University, Via della Vasca Navale 79, 00146 Roma, Italy; ∥ Diamond Light Source, Harwell Science and Innovation Campus, Didcot OX11 0DE, U.K.; ⊥ Department of Pharmaceutical and Pharmacological Sciences, University of Padova, via Marzolo 5, 35131 Padova, Italy; # Department of Industrial Engineering, University of Padova, via Marzolo 9, 35131 Padova, Italy

## Abstract

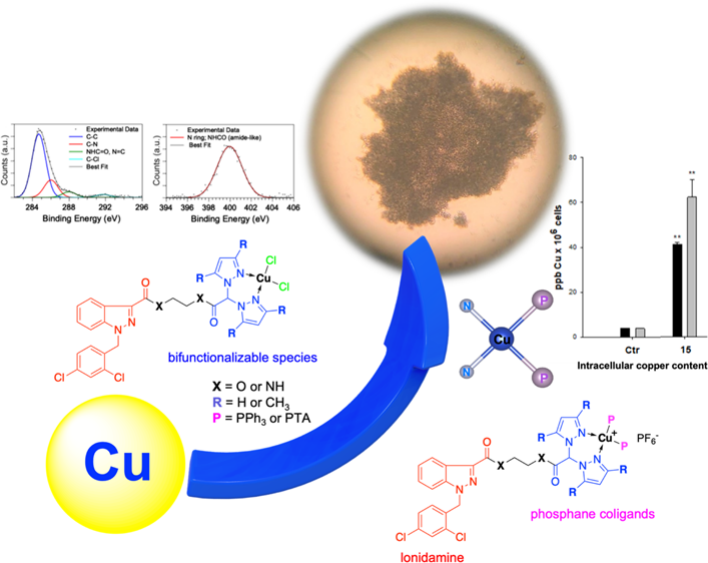

Bis(pyrazol-1-yl)- and bis(3,5-dimethylpyrazol-1-yl)-acetates were conjugated with the 2-hydroxyethylester and 2-aminoethylamide derivatives of the antineoplastic drug lonidamine to prepare Cu(I) and Cu(II) complexes that might act through synergistic mechanisms of action due to the presence of lonidamine and copper in the same chemical entity. Synchrotron radiation-based complementary techniques [X-ray photorlectron spectroscopy and near-edge X-ray absorption fine structure (NEXAFS)] were used to characterize the electronic and molecular structures of the complexes and the local structure around the copper ion (XAFS) in selected complexes. All complexes showed significant antitumor activity, proving to be more effective than the reference drug cisplatin in a panel of human tumor cell lines, and were able to overcome oxaliplatin and multidrug resistance. Noticeably, these Cu complexes appeared much more effective than cisplatin against 3D spheroids of pancreatic PSN-1 cancer cells; among these, PPh_3_-containing Cu(I) complex **15** appeared to be the most promising derivative. Mechanistic studies revealed that **15** induced cancer cell death by means of an apoptosis-alternative cell death.

## Abstract 2

The antineoplastic drug lonidamine was conjugated with bis(pyrazolyl)acetates to obtain new Cu(I) and Cu(II) complexes, whose electronic and molecular structures were investigated using SR-XPS and XAS. All complexes showed significant antitumor activity in human tumor cell lines and were more effective than cisplatin against 3D spheroids of PSN-1 cancer cells.

## Introduction

Lonidamine (LND) is an antineoplastic drug able to sensitize tumors to radio-, chemo-, and photodynamic therapy. Although its mechanism of action is not completely clear yet, its use has been reported to affect the metabolic pathways of cancer cells by inhibiting mitochondrial respiration and glycolysis.^1−3^ It has also been suggested that LND induces intracellular tumor acidification by inhibiting the efflux of l-lactic acid from cells mediated by monocarboxylate transporters and the mitochondrial uptake of pyruvate mediated by the mitochondrial pyruvate carrier.^4,5^ Moreover, this drug induces a mitochondrial transmembrane potential disruption through a direct effect on the mitochondrial permeability transition pore.^6,7^

Although the antitumor activity of LND as a single agent is limited, this drug has great potential in increasing the efficacy of traditional chemotherapeutic agents, including cisplatin and other platinum-based drugs.^1,8−10^ Platinum complexes conjugated with LND or its derivatives showed interesting antitumor activity profiles in vitro, with improved cytotoxic effects compared to that of cisplatin and other reference drugs.^11−13^ In addition, a recent and promising approach concerned the preparation and biological study of gold nanoparticles conjugated with LND and aptamer AS1411 as effective cancer treatments.^14^

Cu(I) and Cu(II) complexes have received great attention for their both in vitro and in vivo unique properties*.*^15−27^ Copper complexes and copper-based nanomedicines^28,29^ are coming out as promising antitumor agents due to the elevated need for copper in cancer tissues compared to that in normal cells and its role as a limiting factor for multiple aspects of tumor progression, including angiogenesis, growth, and metastasis.^22,30−34^ While tumor cells, avid of copper to fuel neovascularization in tumor progression, enrich their copper content, eventually causing copper overload and thus cell death, normal cells continue to adopt physiological mechanisms that regulate copper intracellular concentration. Somewhat reversing the anticancer strategy based on sequestration of copper to prevent establishment of the tumor blood supply,^35^ tumor cells may represent a suitable, selective target for copper-based antitumor drugs.^36^ Actually, anticancer copper-based drugs are endowed with improved selectivity toward tumor cells. Even if little information is available on the molecular basis for the mode of action of copper complexes, several molecular effectors and signalling pathways have emerged as suitable targets for copper complexes.^36,37^

In addition, endogenous metal ions are generally less toxic than nonendogenous ones toward normal cells, and for this reason, copper complexes might represent efficacious alternatives to Pt-based drugs.^34,36,38−42^ Copper complexes show broader spectra of activities and lower toxicity, thereby providing the possibility of circumventing the problems encountered by platinum drugs, such as dose-limiting toxicity and inherent/acquired resistance.^43,44^ Considering their reactivity to biomolecules other than DNA, there is increasing evidence that the mechanisms of action of copper complexes are markedly different from those of platinum drugs.^45,46^ In this contest, some classes of copper complexes were found to exert an effective antiproliferative action by dysregulating mitochondrial function in cancer cells.^47^

To our knowledge, no copper complexes with LND derivatives have been reported in the literature to date. Therefore, the aim of this work was to functionalize LND with species able to coordinate metals in order to form Cu(I) and Cu(II) complexes potentially capable of exerting an anticancer activity through synergistic mechanisms of action.

**Scheme 1:**
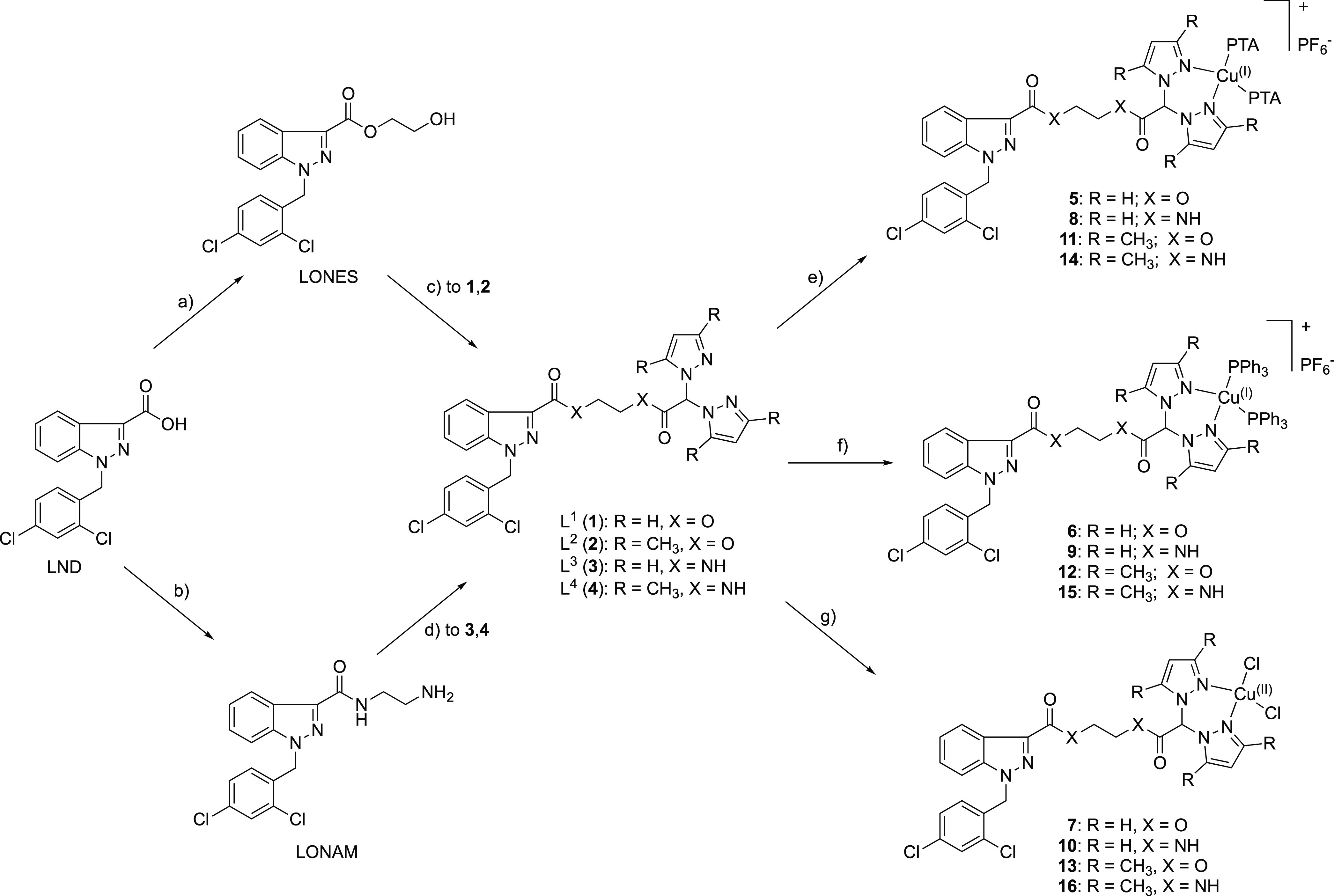
Synthesis of Ligands **1**–**4** and Complexes **5**–**16**; Reagents and Conditions: (a) HOCH_2_CH_2_OH, H_2_SO_4_, 85 °C, 1 h; (b) H_2_NCH_2_CH_2_NH_2_, CDI, THF, 18 h; (c) HC(pz)_2_COOH or HC(3,5-Me_2_pz)_2_COOH, DMAP, EDCI·HCl,16 h; (d) HC(pz)_2_COOH or HC(3,5-Me_2_pz)_2_COOH, HOBT, EDCI·HCl, DMF, 16 h; (e) PTA, Cu(CH_3_CN)_4_PF_6_, CH_3_CN, Overnight; (f) PPh_3_, Cu(CH_3_CN)_4_PF_6_, CH_3_CN, Overnight; and (g) CuCl_2_·2H_2_O, CH_3_CN, 24 h

For this purpose, LND was converted into 2-hydroxyethylester and 2-aminoethylamide derivatives LONES and LONAM, respectively, which were conjugated to bifunctional species bis(pyrazol-1-yl)acetic acid [HC(pz)_2_COOH] and bis(3,5-dimethyl-pyrazol-1-yl)acetic acid [HC(3,5-Me_2_pz)_2_COOH] to form heteroscorpionate ligands **1–4** (L^1^–L^4^, Scheme 1). HC(pz)_2_COOH and HC(3,5-Me_2_pz)_2_COOH were selected as coordinating agents for their κ^3^-NNO coordination properties of bis(azol-1-yl)methane^48^ and for the presence of a carboxylic function suitable to be derivatized with the hydroxyl group of LONES or the primary amino group of LONAM. In addition, we have recently reported that copper complexes with heteroscorpionate ligands, obtained via conjugation with nitroimidazole, glucosamine, and a noncompetitive NMDA receptor antagonist, showed cytotoxic activity toward a panel of several human tumor cell lines.^49−52^

Ligands **1**–**4** were used for the preparation of Cu(I) and Cu(II) complexes **5**–**16** (Scheme 1) that might act through synergistic mechanisms of action due to the presence of LND and copper in the same chemical entity. Concerning the Cu(I) complexes, to stabilize copper in the +1 oxidation state, lipophilic triphenylphosphine (PPh_3_) and hydrophilic 1,3,5-triaza-7-phosphaadamantane (PTA) were selected as coligands in order to confer different solubility properties to the corresponding complexes.

The molecular structure of selected coordination compounds was investigated in the solid state by means of synchrotron radiation-induced X-ray photoelectron spectroscopy (SR-XPS), near-edge X-ray absorption fine structure (NEXAFS), and X-ray absorption spectroscopy (XAS); the multitechnique approach allowed us to properly define the coordination geometry around the copper ion, as well as to ascertain the molecular structural stability of the ligands upon interaction with the metal.^53^

The new complexes **5**–**16**, the corresponding uncoordinated ligands **1**–**4**, and LND were investigated for their cytotoxic potential on a panel of human cancer cell lines, derived from different solid tumors, by means of both 2D and 3D cell viability studies. The cell panel also includes cancer cells selected for their resistance to oxaliplatin or multidrug resistant (MDR) cells. Furthermore, mechanistic studies were performed in order to elucidate the multimodal mechanistic effect of the new Cu(I) and Cu(II) species.

## Results and Discussion

### Synthesis and Characterization

Starting materials LONES and LONAM and ligands L^1^, L^2^, L^3^, and L^4^ were prepared according to the procedure reported in Scheme 1. In particular, the reaction between LND and ethylene glycol in the presence of sulfuric acid led to intermediate LONES, which was treated with acids HC(pz)_2_COOH and HC(3,5-Me_2_pz)_2_COOH^54,55^ in the presence of 3-(ethyliminomethylideneamino)-*N*,*N*-dimethyl-propane-1-amine hydrochloride (EDCI·HCl) and *N*,*N*-dimethylaminopyridine (DMAP) to give ligands L^1^ (**1**) and L^2^ (**2**), respectively. Treatment of LND with ethylenediamine in the presence of carbonyldiimidazole (CDI) led to intermediate LONAM, which reacted with HC(pz)_2_COOH or HC(3,5-Me_2_pz)_2_COOH in the presence of EDCI·HCl and 1-hydroxybenzotriazole (HOBT) to give ligands L^3^ (**3**) and L^4^ (**4**), respectively. After separation and purification via column chromatography, ligands **1**–**4** were obtained in a reasonable yield and purity. They are soluble in CHCl_3_, CH_2_Cl_2_, dimethyl sulfoxide (DMSO), and CH_3_OH but insoluble in water. Ligands **1**–**3** are also soluble in CH_3_CN. The infrared (IR) spectra obtained for solid samples of ligands **1**–**4** showed all the bands expected for these heteroscorpionate ligands. The ^1^H nuclear magnetic resistance (NMR) spectra, recorded in CDCl_3_ and in DMSO-*d*_6_ solution at room temperature, show all the expected signals for the bioconjugated ligands with a single set of resonances for the pyrazole rings, indicating that the pyrazole protons are equivalents. The elemental analyses confirm the stoichiometry and the purity of the products in the solid state.

Cu(I) complexes [(PTA)_2_Cu(L^1^)]PF_6_ (**5**), [(PTA)_2_Cu(L^3^)]PF_6_ (**8**), [(PTA)_2_Cu(L^2^)]PF_6_ (**11**), and [(PTA)_2_Cu(L^4^)]PF_6_ (**14**) were prepared via the reaction of PTA, Cu(CH_3_CN)_4_PF_6_, and ligands L^1^, L^3^, L^2^, and L^4^, respectively (Scheme 1), following a one-pot synthesis with CH_3_CN as the solvent. Analogously, Cu(I) complexes [(PPh_3_)_2_Cu(L^1^)]PF_6_ (**6**), [(PPh_3_)_2_Cu(L^3^)]PF_6_ (**9**), [(PPh_3_)_2_Cu(L^2^)]PF_6_ (**12**), and [(PPh_3_)_2_Cu(L^4^)]PF_6_ (**15**) were prepared via the reaction of PPh_3_, Cu(CH_3_CN)_4_PF_6_, and the related ligands (Scheme 1). All the compounds are soluble in CH_3_CN and DMSO; the complexes with PPh_3_ coligands (**6**, **9**, **12**, and **15**) are also soluble in CHCl_3_; complexes **6**, **9**, **12**, and **14** are soluble in methanol; complex **15** is soluble in ethanol; while complexes **8**, **9**, and **11** are soluble in acetone. The IR spectra obtained for solid samples of the Cu(I) complexes show all the expected bands for the bioconjugated ligands and the phosphane coligands. The absorptions due to the C=O stretching of the ester groups for complexes **5**, **6**, **11**, and **12** do not significantly vary with respect to the same absorptions of the carbonyl groups detectable in the spectra of the free ligands. The absorptions due to the C=O stretching of the amide groups for **8**, **9**, **14**, and **15** are slightly shifted at lower frequencies with respect to those of the free ligands. The ^1^H NMR spectra of the Cu(I) complexes, recorded in DMSO-*d*_6_ solution at room temperature, showed a single set of resonances for the pyrazole rings, indicating that the pyrazole protons are equivalents, with a slight shift due to the coordination to the metal center. A significant shift to higher frequencies of the N–*H* signals is detected only for compounds **14** (δ 8.43 and 9.07 ppm) and **15** (δ 8.38 and 9.35 ppm) probably due to a secondary interaction between the hydrogen atoms and the copper center. The PTA and PPh_3_ coligands show a characteristic series of peaks at δ 4.04–4.73 and 7.20–7.80 ppm, respectively, with an integration, with respect to the ligand peaks, that confirms the 1:2 stoichiometric ratio between the ligand and the phosphane coligands. The room-temperature ^31^P{H} NMR spectra of the Cu(I) complexes, recorded in DMSO-*d*_6_ and CD_3_CN solution at room temperature, give singlets shifted downfield with respect to the value of the free phosphanes PPh_3_ and PTA. The characteristic septet centered at about δ −144 ppm is due to the presence of the PF_6_^–^ counterion. The electrospray ionization mass spectroscopy (ESI-MS) studies, performed by dissolving the Cu(I) complexes in CH_3_CN and recording the spectra in the positive- and negative-ion modes, confirm the formation of the PTA and PPh_3_ complexes and the presence of hexafluorophosphate as counterions.

Cu(II) complexes [(L^1^)CuCl_2_] (**7**), [(L^3^)CuCl_2_] (**10**), [(L^2^)CuCl_2_] (**13**), and [(L^4^)CuCl_2_] (**16**) were prepared via the reaction of CuCl_2_·2H_2_O with ligands L^1^, L^3^, L^2^, and L^4^, respectively, in acetonitrile solution for **7** and **13** and in methanol solution for **10** and **16** at room temperature (Scheme 1). All the compounds are soluble in DMSO; complexes **7** and **13** are soluble in CH_3_OH, CHCl_3_, and CH_3_CN; complexes **10** and **16** are slightly soluble in CH_3_CN; complex **16** is soluble in CHCl_3_; and complexes **13** and **16** are soluble in CH_2_Cl_2_. The IR spectra obtained for solid samples show all the expected bands for the bioconjugated ligands. The strong absorptions due to the C=O stretching of the ester and amide groups do not significantly vary with respect to the absorptions detectable for the free ligands. These data indicate that the carbonyl groups are not involved in the coordination of the metal: the copper center results in a tetracoordinated environment with the ligand chelating in a bidentate fashion and the other two positions being occupied by the chlorides. The ESI-MS studies, conducted by dissolving the Cu(II) complexes in CH_3_CN and recording the spectra in the positive- and negative-ion modes, confirm the formation of the complexes and the presence of the chlorides as counterions.

### Investigation of the Molecular and Electronic Structures

#### Synchrotron Radiation-Induced X-ray Photoelectron Spectroscopy

The electronic and molecular structures of coordination compounds **10**, **15**, and **16**, in comparison with those of ligand **4**, were probed using SR-XPS. SR-XPS spectra were collected at C 1s, N 1s, O 1s, Cl 2p, P 2p (for **15**), F 1s (for **15**), and Cu 2p core levels; the detailed data analysis results [binding energy (BE), full width at half maximum (FWHM), and assignments], confirming the proposed molecular structures for the complexes and the stability of the ligand **4** molecular structure upon coordination to copper, are collected in the Supporting Information (Table S1). In the following, the most interesting signals are described and compared for the three analyzed samples, considered representative of Cu(II) and Cu(I) coordination compounds.

**Figure 1:**
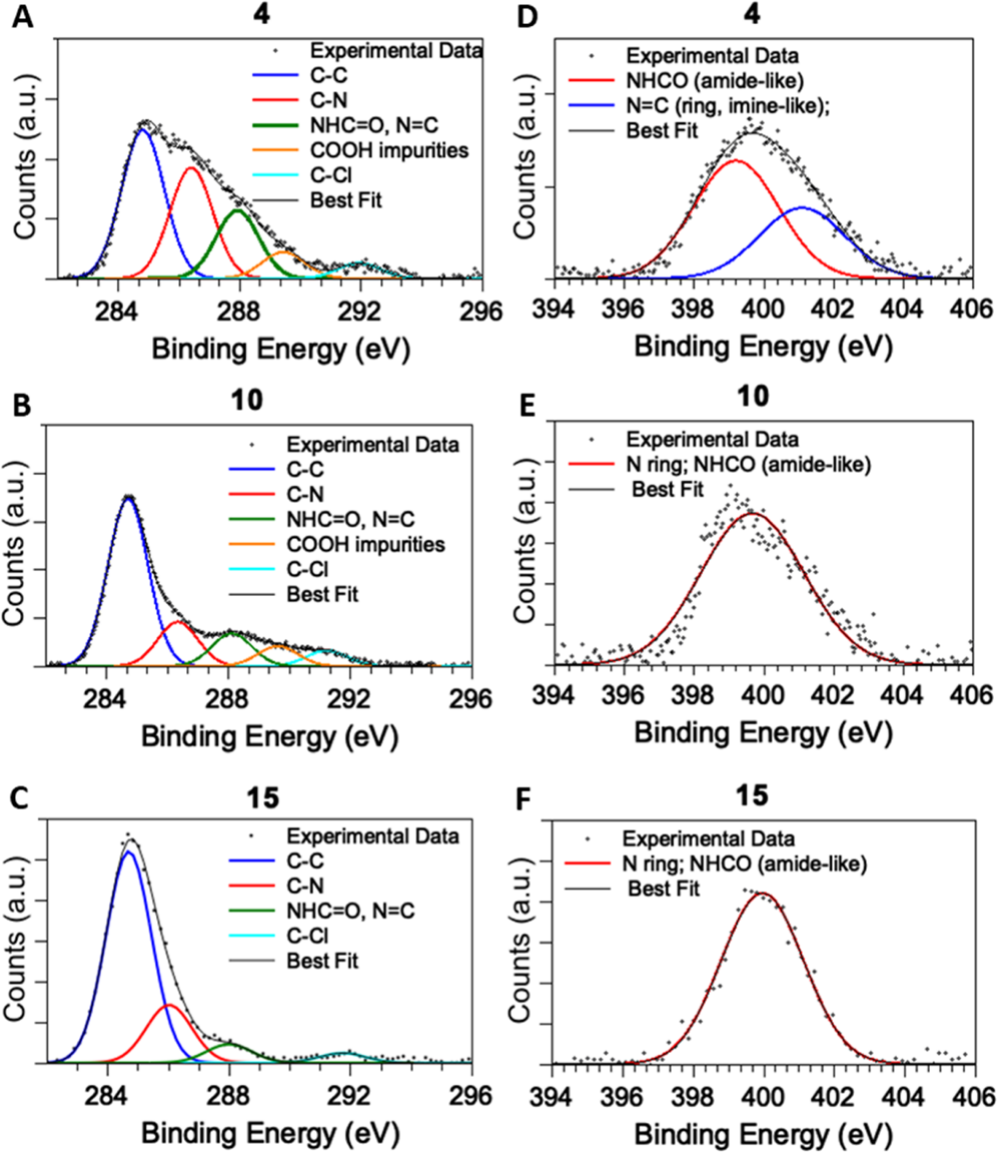
C 1s spectra of ligand **4** (A), Cu(II) complex **10** (B), and Cu(I) complex **15** (C) and N 1s spectra of **4** (D) and coordination compounds **10** (E) and **15** (F).

The C 1s signal can always be resolved using curve fitting analysis into several components corresponding to the different C atoms in the proposed molecular structure. More in detail, in the order of increasing BE, the contributions are assigned as follows: aromatic and aliphatic C–C carbons (BE = 284.7 eV), C–N carbons of the pyrazole-like rings (BE = 286.6 eV), C=O carbonyls of amide groups and imine-like C=N groups (BE = 288.0 eV), −COOH impurities always found on the surface of samples deposited in air (BE = 289.3 eV), and C–Cl groups (BE = 291.5 eV). C 1s spectra of ligand **4** and complexes **10** and **15** are reported in Figure 1A–C.

N 1s spectra of ligand **4** and complexes **10** and **15** are reported in Figure 1D–F, respectively. For **4**, a couple of main signals can be detected at 399.5 and 401.0 eV BEs, indicative for the two kinds of nitrogens of the pyrazole rings, that is, amine-like and imine-like N atoms, respectively.^56^ Amide-like N atoms are found at a BE value very close to that of the amine-like N atoms and cannot be resolved due to the experimental resolution (0.6 eV). As for the coordination compounds, it is expected that only the amine-like contribution appears when the two nitrogen atoms coordinate with a metal ion, as reported in the literature for heterocycles coordinating metal ions (e.g., porphyrins or phthalocyanines).^57−59^ In excellent agreement with this prediction, in complexes **10**, **15** (Figure 1E,F, respectively), and **16**, a single N 1s component at about 400.0 eV, attributed to the symmetrized nitrogen atoms coordinating to copper ions (and to the indistinguishable amide-like N), can be observed. Cl 2p BE values observed for both ligand **4** and the three coordination compounds **10**, **15**, and **16** (Cl 2p_3/2_ BE = 200 eV) are compatible with chlorine atoms covalently bonded to carbon in organic molecules.^60^ In addition, in Cl 2p spectra of **10** and **16**, a contribution of slightly higher intensity at a lower BE (198 eV BE) can be observed, as expected for chlorine atoms bonded to Cu(II) ions in the coordination compounds.^60,61^

Cu 2p spectra collected for copper complexes **10** and **15** are reported in Figure S1 in the Supporting Information; both Cu 2p spectra collected for complexes **10** and **16** show a spin–orbit pair with the Cu 2p_3/2_ component centered at 936 eV, indicative of Cu(II) ions in coordination compounds,^60^ in excellent agreement with analogous systems.^59^ On the other hand, the Cu 2p spectrum collected on complex **15** has the Cu 2p_3/2_ spin–orbit component centered at a 932.0 eV BE, as expected for Cu(I) ions in coordination compounds.^60^

#### NEXAFS Data Analysis Results

**Figure 2:**
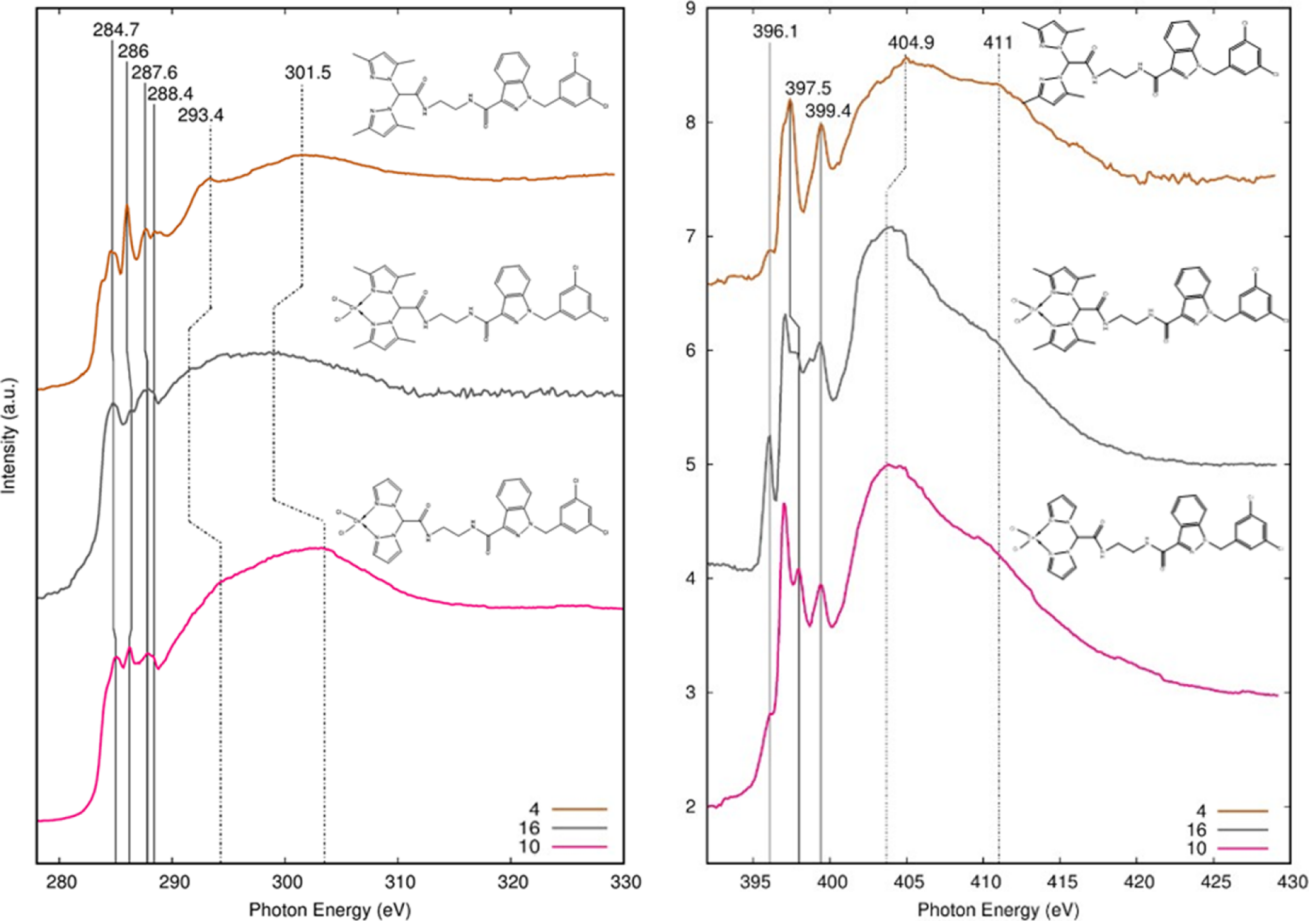
C K-edge (left) and N K-edge (right) NEXAFS spectra of ligand **4** and Cu(II) complexes **10** and **16**.

NEXAFS spectroscopy measurements were carried out at C and N K-edges on ligand **4** and on coordination compounds **10** and **16**, with the aim to obtain further information about the influence of the metal coordination on the electronic structure of the ligand. Experimental spectra of the C K-edge and N K-edge of **4**, **10**, and **16** samples are reported in Figure 2. They were collected at the grazing incidence of the polarized photon beam with respect to the sample surface; no angular dependence was observed on the NEXAFS spectra of the investigated compounds when the incidence angle of the impinging radiation was changed from grazing to magic and normal, indicating the absence of a preferential orientation of the investigated molecules on the sample surface.

According to the literature,^59,62−65^ peak positions and assignments of the main features detected in the C and N K-edge spectra of the analyzed samples are also shown in Table 1.

**Table 1:** NEXAFS: Peak Position (eV) and Relative Assignment of the Main Features Appearing in the C and N K-Edge NEXAFS Spectra of Samples **4**, **10**, and **16**

sample	**4**	**10**	**16**	assignment
C K-edge	284.7	285.1	284.8	π_C=C_^*^
	286.0	286.2	286.4	π_C=N_^*^
	287.6	287.8	287.8	σ_C–H_^*^
	288.4	288.4	288.4	π_C=O_^*^
	293.4	294.3	291.5	σ_C–C_^*^
	301.5	303.5	299.0	σ_C=N_^*^
N K-edge	396.1	396.1	396.1	
	397.5	397.9	397.9	π_1_^*^
	399.4	399.4	399.4	π_2_^*^
	404.9	403.7	403.7	σ_C=N_^*^
	411.0	411.0	411.0	σ_C–N_^*^

For the C K-edge spectra, the energy scale is referenced to the π_C=O_^*^ transition of the amide function in the side chain of LND,^62,65^ while for the N K-edge spectra, the energy scale is referenced to the π_2_^*^ transition of the pyrazole ring.^59,63,64^

The C K-edge spectra present the expected π_C=C_^*^ and π_C=N_^*^ features of the pyrazole ring and the π_C=O_^*^ feature related to the amide group at 288.4 eV. It can be noticed (Table 1) that the first two peaks lie at lower energy in **4** and that the π_C=N_^*^ peak is attenuated in Cu complexes **10** and **16** with respect to that in ligand **4**. These effects could be related to the Cu complexation of the pyrazole nitrogens.

All the samples exhibit a σ_C–H_^*^ resonance originated from the presence of the aliphatic chains. Above the edge, two large features σ_C–C_^*^ and σ_C=N_^*^ can be observed.

The N K-edge spectra show the N 1s → π* transitions (π_1_^*^ and π_2_^*^) originating from two distinct nitrogen atoms. As shown in Table 1, the energy of the π_1_^*^ peak is higher for Cu complexes **10** and **16** than for ligand **4**: this effect might be further evidence of the complexation with copper. The weak feature detected at 296.1 eV for all the analyzed samples is probably related to impurities on the beamline mirrors.

#### XAFS Data Analysis Results

**Figure 3:**
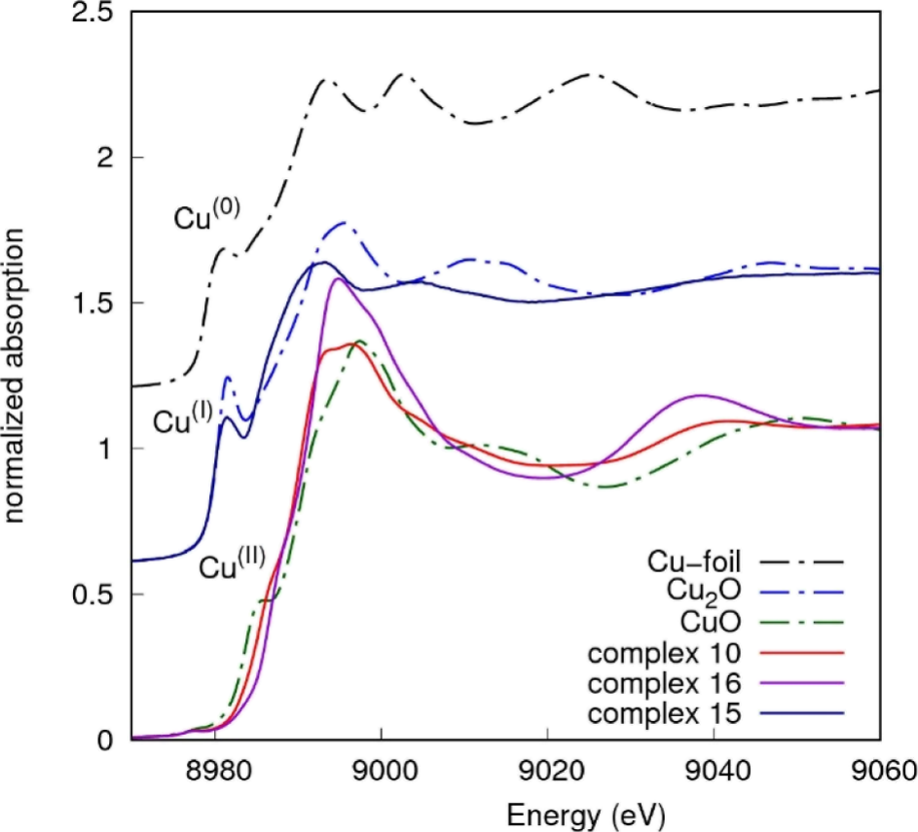
Cu K-edge normalized XANES spectra measured on complexes and reference compounds, shifted for the sake of clarity. Edge energies of Cu(II) complexes **10** and **16** match the edge energy of the CuO reference compound. The edge energy of Cu(I) complex **15** matches the edge energy of Cu_2_O.

X-ray absorption data collected at the Cu K-edge on selected complexes **10**, **15**, and **16** were analyzed with the aim of understanding the average local coordination chemistry and electronic structure around Cu. The main near-edge features (XANES) originate from the absorber valence state (edge position) and coordination geometry (edge shape).^66^ The Cu K-edge normalized XANES spectra measured on complexes **10**, **15**, and **16** are presented in Figure 3, together with those measured on Cu metal foil and reference Cu oxides, for the sake of comparison. The edge energies of complexes **10** and **16** match the edge energy of the pure CuO reference compound, in accordance to the Cu(II) valence state in these complexes.^59^ A roughly planar geometry could be assumed for sample **10** due to the close similarity with the XANES spectrum of the glycine complex reported in the literature.^67^ The XANES spectrum measured from complex **16** (Figure 3) depicts a higher white line (around 8995 eV) with respect to complex **10** and attenuation of the pre-edge shoulder, this behavior being compatible with the Cu coordination geometry changing from square planar in complex **10** to nearly octahedral in complex **16**. The complex **15** edge position matches the edge energy measured on the pure Cu_2_O reference compound, confirming the Cu(I) valence state. The pre-edge peak we found at 4 eV above the Cu^0^ edge is related to the copper coordination geometry and type of neighbors. Its amplitude is expected to be the highest (around 1) for the Cu(I) bidentate coordination,^66^ while it decreases upon increasing the Cu(I) coordination number and/or reducing the Cu(I) site symmetry.^68,69^ We found the pre-edge peak amplitude of around 0.52 of the edge jump consistent with 3- or 4-coordinated Cu. The squeezing of the structural oscillations in the XANES region, with respect to the Cu_2_O spectrum, suggests averagely longer neighbor distances in complex **15**. Further details about the local structure are obtained from the quantitative analysis of the EXAFS region.

**Figure 4:**
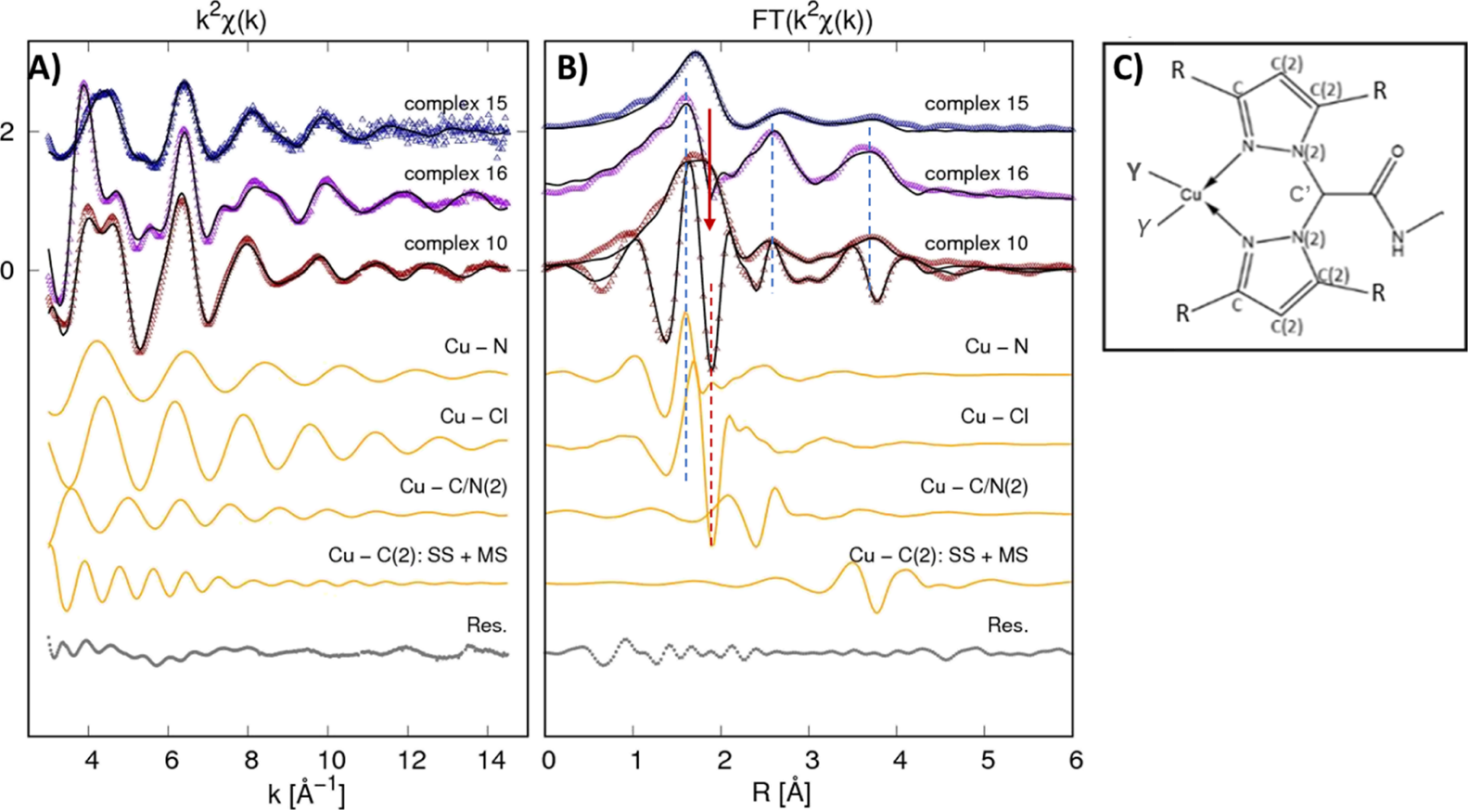
(A) Cu K-edge EXAFS data analysis of complexes **10**, **15**, and **16** are reported. At the top, the experimental *k*^2^χ_exp_(*k*) (dots) and best fit curves *k*^2^χ_fit_(*k*) (black lines) are presented (vertically shifted for clarity). The middle curves (orange) represent the partial contributions used in the analysis of Cu(II) complex **10** (vertically shifted for clarity) for the sake of the example, and the lowest curve (gray) is the best fit residual *k*^2^(χ_fit_ – χ_th_). (B) Corresponding FT moduli are shown for experimental *k*^2^ weighted EXAFS data (dots) and the best fit (black lines). The FT imaginary (Imm-FT) part of the experimental spectrum, best fit, and partial contributions are shown for complex **10** for the sake of an example. The dashed lines highlight in-phase Imm-FT oscillations for Cu–N and Cu–Cl contributions in Cu(II) complexes **10** and **16**. The red arrow points out the structural signal lack in the region of the Cu–Cl shell (red dashed line) likely due to some antiphase structural signals. (C) Local structure around Cu is shown to highlight the neighbor shells involved in the analysis, being R = H (complex **10**) or R = CH_3_ (complex **16**) (see Scheme 1) and Y = Cl [Cu(II) complexes **10** and **16**] and Y = P [Cu(I) complex **15**].

Looking at the *k*^2^-weighted EXAFS signal in the *k* space and at its Fourier transform (FT) in the real space, remarkable differences are evident among the samples (Figure 4). In particular, looking at complexes **10** and **16**, the *k*^2^χ(*k*) signal presents a prominent first oscillation in sample **16**, while in sample **10**, two oscillations of equal and lower intensity are visible. Moreover, from the first oscillation onward, the signals become partially out of phase. Such a different behavior corresponds to differences in the real space for the FT spectra. Noticeably, the modulus and imaginary part of the FT depict very similar shapes (Figure 4, dashed lines) except around 2 Å (Figure 4, red arrow), where a lack of the structural signal is found in the sample **16** data with respect to the sample **10** data. This behavior may point out some antiphase structural signal originating from a different neighbor arrangement around Cu in the two complexes.^70^ Noticeably, complex **16** has two CH_3_ substituents that may provide such an additional coordination shell, in agreement with the XANES features suggesting the nearly octahedral coordination. Moreover, such a different coordination seems to promote greater rigidity of the structure, providing larger next neighbor signals in the next neighbor region (highlighted by dashed lines in Figure 4B). Looking at the weaker *k*^2^χ(*k*) signal of complex **15**, it is evident that the local structure around Cu(I) is definitively more disordered than those of Cu(II) complexes.

Quantitative details were obtained from EXAFS data fitting (see the Supporting Information for details). The distances and mean square relative displacement (MSRD) σ^2^ obtained from the refinement are reported in Table 2 for single scattering (SS) contributions. In complex **10**, the distances demonstrate a good agreement with the expected molecular structure. The Cu–N nearest neighbor shell is found at 1.96 Å, and Cl is at 2.23 Å from Cu(II). The complex **16** EXAFS data analysis demonstrates the octahedral distorted geometry for Cu in complex **16** with two closer Cu–N and Cu–C^R^ nearest neighbor shells and a third longer Cu–Cl shell, and the Cu–C^R^ link would likely originate from the CH_3_ residue. Noticeably, the bimodal Cu–C/N(2) shell resulting in a shorter shell at around 2.9 Å and a long one at around 3.3 Å is consistent with the tilting of pyrazole rings.

**Table 2:** Best Fit Results for Cu K-Edge XAFS Data Analysis of Complexes **10**, **15**, and **16**^a^

		sample **10** Cu(II)		sample **16** Cu(II)		sample **15** Cu(I)	
	N	*R* (Å)	σ^2^ × 10^2^ (Å^2^)	*R* (Å)	σ^2^ × 10^2^ (Å^2^)	*R* (Å)	σ^2^ × 10^2^ (Å^2^)
Cu–N	2	1.959(5)	0.68(5)	1.967(7)	0.24(3)	2.001(3)	3.2(2)
Cu–C^R^	2			1.89(2)	1.62(2)		
Cu–Cl(P)	2	2.23(1)	0.64(4)	2.21(2)	2.3(2)	2.16(1)	0.69(3)
Cu–C/N(2)	4	2.97(3)	0.86(2)	2.85(2)	0.75(3)	2.92(2)	1.3(2)
				3.35(3)	0.45(2)		
Cu–C^Ph3^	9					3.29(3)	1.4(5)
Cu–C(2) (SS + MS)	4	4.08(3)		4.40(3)	0.8	4.05(8)	2.1

a The Cu–C^R^ shell originates from the carbons of R = CH_3_ groups of the pyrazoles; Cu–C^Ph_3_^ originates from the C atoms of phenyl rings bonded to P of PPh_3_. The multiplicity numbers (N) are constrained to the structural model, and interatomic distances [*R* (Å)] and the MSRD (σ^2^) parameters are shown. Standard uncertainties on the last digit are reported in parentheses.

In complex **15**, the Cu–N nearest neighbor distance is slightly longer with respect to the other complexes and largely more disordered. We found a significatively better fit using 2N + 2P neighbor shells with respect to 1N + 2P or 2N + 1P, but the quite large σ^2^ found for the Cu–N suggests more loosely bound Cu(I) in complex **15** with respect to complexes **10** and **16**, likely related to the lower electronegativity of Cu(I). In summary, the Cu oxidation and the local structure around Cu obtained via XAFS is consistent with the expected complex structure; the Cu(I) local structure appears more distorted likely due to the more loosely bound neighbor.

#### Stability Studies

The stability of the new complexes in 0.5% DMSO/RPMI cell culture medium was also evaluated using UV–vis spectroscopy. Changes observed in the UV–vis spectra of the complexes over 24 h were insignificant or only minimal for complexes with L^3^ and L^4^ ligands, indicating that these complexes are stable under physiological conditions (Figure S2 in the Supporting Information). On the contrary, for complexes with L^1^ and L^2^ ligands, a more evident change in the UV–vis spectrum was detected.

#### Cytotoxicity Studies

The newly developed complexes **5**–**16**, the corresponding uncoordinated ligands **1**–**4**, and their precursors were evaluated for their ability to promote cell death in a panel of human cancer cell lines derived from solid tumors (2008 ovarian, HCT-15 colon, PSN-1 pancreatic, A431 cervical, and H157 lung carcinoma cells). The cytotoxicity parameters, expressed in terms of IC_50_ and obtained after 72 h of drug exposure using a 3-[4,5-dimethylthiazol-2-yl]-2,5 diphenyl tetrazolium bromide (MTT) assay, are reported in Table 3. For comparison purposes, the cytotoxicity of cisplatin was assessed under the same experimental conditions.

**Table 3:** Cytotoxic Activity of LND, LONES, LONAM, **1**–**16**, and Cisplatin^a^

	IC_50_ (μM) ± S.D.				
compound	2008	HCT-15	PSN-1	H157	A431
LND	24.9 ± 3.3	>25	24.6 ± 2.9	>25	>25
LONES	>25	>25	21.3 ± 2.4	>25	>25
LONAM	18.2 ± 0.9	>25	>25	>25	>25
**1** (L^1^)	11.70 ± 0.02	13.4 ± 5.3	14.3 ± 3.8	16.8 ± 2.4	12.5 ± 2.1
**2** (L^2^)	21.3 ± 3.5	6.2 ± 0.5	2.7 ± 0.3	5.2 ± 0.7	3.2 ± 0.2
**3** (L^3^)	7.9 ± 2.2	4.7 ± 1.6	3.1 ± 0.9	5.6 ± 1.4	3.7 ± 1.6
**4** (L^4^)	2.9 ± 1.1	1.9 ± 1.0	1.5 ± 0.5	1.3 ± 0.6	1.4 ± 0.5
**5** [(PTA)_2_Cu(L^1^)][PF_6_]	2.4 ± 0.9	2.5 ± 0.5	1.2 ± 0.1	1.3 ± 0.5	0.4 ± 0.1
**6** [(PPh_3_)_2_Cu(L^1^)][PF_6_]	2.2 ± 0.8	1.7 ± 0.6	1.4 ± 0.1	1.30 ± 0.03	0.6 ± 0.3
**7** [(L^1^)CuCl_2_]	2.8 ± 0.9	1.6 ± 0.4	1.6 ± 0.4	0.6 ± 0.2	0.70 ± 0.02
**8** [(PTA)_2_Cu(L^3^)]PF_6_	0.8 ± 0.3	0.4 ± 0.1	0.6 ± 0.2	0.6 ± 0.1	0.60 ± 0.01
**9** [(PPh_3_)_2_Cu(L^3^)]PF_6_	1.1 ± 0.3	0.30 ± 0.01	0.5 ± 0.1	0.40 ± 0.01	1.0 ± 0.2
**10** [(L^3^)CuCl_2_]	1.20 ± 0.02	0.2 ± 0.1	0.5 ± 0.2	0.5 ± 0.2	1.6 ± 0.8
**11** [(PTA)_2_Cu(L^2^)][PF_6_]	2.2 ± 0.9	2.2 ± 0.4	1.0 ± 0.4	0.5 ± 0.2	0.6 ± 0.1
**12** [(PPh_3_)_2_Cu(L^2^)][PF_6_]	1.4 ± 0.1	3.4 ± 0.1	0.6 ± 0.1	1.2 ± 0.2	0.7 ± 0.1
**13** [(L^2^)CuCl_2_]	1.3 ± 0.2	4.7 ± 0.9	0.5 ± 0.1	1.0 ± 0.2	1.5 ± 0.6
**14** [(PTA)_2_Cu(L^4^)]PF_6_	0.7 ± 0.2	0.3 ± 0.1	1.0 ± 0.3	0.900 ± 0.001	0.5 ± 0.2
**15** [(PPh_3_)_2_Cu(L^4^)]PF_6_	0.6 ± 0.1	0.3 ± 0.1	0.6 ± 0.1	0.3 ± 0.1	0.4 ± 0.2
**16** [(L^4^)CuCl_2_]	0.8 ± 0.8	0.2 ± 0.1	1.6 ± 0.2	0.60 ± 0.02	1.00 ± 0.01
cisplatin	2.2 ± 1.0	15.3 ± 2.6	12.1 ± 2.8	2.1 ± 0.8	2.1 ± 0.9

a Cells (3–8 × 10^3^ mL^–1^) were treated for 72 h with the tested compounds. Cell viability was measured by means of an MTT test. The IC_50_ values were calculated using a 4-PL logistic model (*P* < 0.05). S.D. = standard deviation.

LND and ligand precursors LONAM and LONES did not induce a significant reduction of cell viability (the IC_50_ values were greater than 25 μM). Uncoordinated ligands **1**–**4** possessed a moderate cytotoxic potency that was, however, on average, 3 to 10 times lower than that of the corresponding metal complexes. Actually, all tested copper complexes showed a promising cytotoxic potential, with IC_50_ values in the low or submicromolar range toward all the human cancer cell lines belonging to the in-house panel, and proved to be more effective than the reference chemotherapeutic drug cisplatin. These results suggest that both LND and copper might contribute to the potent antitumor activity of these complexes.

In general, L^1^ and L^2^ derivatives were on average less effective than the corresponding L^3^ and L^4^ complexes, and among the series, no significant differences in terms of in vitro antitumor activities were detected for Cu(I) and Cu(II) complexes. It is important to note that complexes with L^1^ and L^2^ ligands were less stable in physiological media compared with complexes bearing L^3^ and L^4^. Hence, their lower cytotoxic effectiveness could be attributed, at least in part, to their instability in physiological conditions. Among all, complex **15** was the most effective derivative, eliciting, on average, IC_50_ values about 16 times lower than those detected with cisplatin. On the contrary, compound **5** was the weakest among the series, with a cytotoxic potency that was, however, more than 4 times higher than that of cisplatin.

Considering the very promising antiproliferative effects and keeping in mind that drug resistance represents a key determinant for the variable efficacy of anticancer therapy, we also assessed the ability of the newly developed Cu(I) and Cu(II) complexes to bypass the acquired drug resistance. In particular, complexes were evaluated for their antiproliferative activity against some cancer cell lines selected for sensitivity/resistance to oxaliplatin or MDR cells, namely, LoVo, LoVo-OXP, and LoVo MDR human colon cancer cells.

As previously reported, LoVo OXP cells (derived from LoVo cells grown in the presence of increasing concentrations of oxaliplatin) were about 13-fold more resistant to oxaliplatin than parental cells.^23^ The main molecular mechanisms involved in oxaliplatin resistance appear to depend upon (i) the decreased cellular accumulation, which is thought to be related to a greater activity of the ATP7B exporter rather than the activity of P-glycoprotein (P-gp) and multidrug resistance protein 1 (MRP1), and (ii) the more efficient repair of oxaliplatin-induced DNA damage via nucleotide excision repair (NER).^71^ Conversely, in human colon LoVo MDR cancer cells, the resistance to doxorubicin, a drug belonging to the MDR spectrum, is associated with an overexpression of drug transporters, such as the 170 kDa P-gp.^72^

Table 4 shows the degree of resistance in terms of the resistant factor (RF), which is defined as the ratio between IC_50_ (obtained using an MTT assay after 72 h of drug exposure) calculated for the resistant cells and those arising from the sensitive ones (Table 4).

**Table 4:** Cross-Resistance Profile of Complexes **5**–**16**, Oxaliplatin, and Doxorubicin^a^

	IC_50_ (μM) ± S.D.				
compound	LoVo	LoVo OXP	RF	LoVo MDR	RF
**5** [(PTA)_2_Cu(L^1^)][PF_6_]	2.4 ± 0.3	1.8 ± 0.5	0.8	1.6 ± 0.7	0.7
**6** [(PPh_3_)_2_Cu(L^1^)][PF_6_]	1.4 ± 0.4	1.1 ± 0.3	0.8	1.2 ± 0.4	0.9
**7** [(L^1^)CuCl_2_]	1.8 ± 0.5	1.7 ± 0.6	0.9	1.0 ± 0.4	0.6
**8** [(PTA)_2_Cu(L^3^)]PF_6_	0.9 ± 0.1	0.7 ± 0.2	0.8	0.7 ± 0.3	0.9
**9** [(PPh_3_)_2_Cu(L^3^)]PF_6_	0.20 ± 0.05	0.30 ± 0.04	1.5	0.10 ± 0.03	0.5
**10** [(L^3^)CuCl_2_]	0.6 ± 0.1	0.7 ± 0.1	1.0	0.8 ± 0.2	1.3
**11** [(PTA)_2_Cu(L^2^)][PF_6_]	2.8 ± 0.7	2.2 ± 0.9	0.8	2.5 ± 0.4	0.9
**12** [(PPh_3_)_2_Cu(L^2^)][PF_6_]	2.2 ± 0.4	1.6 ± 0.3	0.7	1.7 ± 0.6	0.8
**13** [(L^2^)CuCl_2_]	2.8 ± 0.7	2.1 ± 0.6	0.8	2.0 ± 0.5	0.7
**14** [(PTA)_2_Cu(L^4^)]PF_6_	0.4 ± 0.1	0.7 ± 0.1	1.8	0.7 ± 0.2	1.0
**15** [(PPh_3_)_2_Cu(L^4^)]PF_6_	0.20 ± 0.02	0.20 ± 0.03	1.0	0.10 ± 0.01	0.5
**16** [(L^4^)CuCl_2_]	0.8 ± 0.1	0.9 ± 0.3	1.1	1.1 ± 0.3	1.4
oxaliplatin	1.5 ± 0.6	19.6 ± 1.9	13.1		
doxorubicin	1.1 ± 0.5			19.4 ± 2.2	17.4

a Cells (5 × 10^3^ mL^–1^) were treated for 72 h with the tested compounds. Cell viability was measured by means of an MTT test. IC_50_ values were calculated using the 4-PL logistic model (*P* < 0.05). S.D. = standard deviation. RF = IC_50_ (resistant cells)/IC_50_ (wild-type cells).

All complexes were equally effective against sensitive (LoVo) and resistant (LoVo-OXP) colon cancer cells and possessed RFs much lower than that of doxorubicin, thus attesting their ability to overcome the oxaliplatin resistance and the MDR phenomenon and not acting as P-gp substrates.

Clearly, such results make these complexes promising for further biological studies aiming at an application in solid tumors refractory to platinum drug treatment.

In an attempt to better appreciate the antitumor potential of the new Cu(I) and Cu(II) complexes containing the LND-conjugated ligands, we compared their cytotoxic profiles with those of similarly unconjugated or differently conjugated (pyrazolyl)acetate complexes that we had previously characterized.^49,52,73,74^ No significant differences in terms of antiproliferative activity and overcoming drug resistance have been detected. The calculated IC_50_ values, both in sensitive and resistant cancer cells, were always in the very low or submicromolar range. The newly developed copper complexes were also screened against 3D spheroids of pancreatic PSN-1 cancer cells to further evaluate their anticancer potential. Actually, 3D cell cultures possess several features that more closely mimic the in vivo tumor architecture and physiology, being consequently potentially more predictive for in vivo effectiveness.^75^ The cancer spheroids were treated with the tested complexes for 72 h, and cell viability was assessed by means of the acid phosphatase (APH) assay (Table 5). Notably, all complexes were much more effective than cisplatin against the 3D model. Similarly to 2D studies, L^1^ and L^2^ derivatives were less effective than the corresponding L^3^ and L^4^ complexes. Differently from cytotoxicity studies performed on 2D cell cultures, in 3D models, Cu(I) complexes with PPh_3_ ligands proved to be much more effective than Cu(II) derivatives. These results could be related to the more lipophilic character of PPh_3_-containing Cu(I) complexes, which makes them more effective at penetrating across the entire spheroid domain, including the inner core. Complex **15** again emerged as the most promising derivative, with an IC_50_ value roughly 12-fold better than that of cisplatin, and complex **5** as the less effective derivative of the series.

**Table 5:** Cytotoxicity toward Pancreatic PSN-1 Cancer Cell Spheroids of Complexes **5**–**16** and Cisplatin^a^

	IC_50_ (μM) ± S.D.
compound	PSN-1
**5** [(PTA)_2_Cu(L^1^)][PF_6_]	40.4 ± 2.0
**6** [(PPh_3_)_2_Cu(L^1^)][PF_6_]	8.3 ± 1.2
**7** [(L^1^)CuCl_2_]	33.0 ± 3.0
**8** [(PTA)_2_Cu(L^3^)]PF_6_	11.6 ± 1.6
**9** [(PPh_3_)_2_Cu(L^3^)]PF_6_	6.8 ± 0.4
**10** [(L^3^)CuCl_2_]	7.6 ± 0.3
**11** [(PTA)_2_Cu(L^2^)][PF_6_]	31.9 ± 4.0
**12** [(PPh_3_)_2_Cu(L^2^)][PF_6_]	22.0 ± 2.1
**13** [(L^2^)CuCl_2_]	34.0 ± 2.1
**14** [(PTA)_2_Cu(L^4^)]PF_6_	6.6 ± 0.5
**15** [(PPh_3_)_2_Cu(L^4^)]PF_6_	4.5 ± 0.4
**16** [(L^4^)CuCl_2_]	13.7 ± 3.7
cisplatin	52.6 ± 4.9

a Cancer cell spheroids (2.5 × 10^3^ cells/well) were treated for 72 h with the tested compounds. Cell viability was evaluated by means of the APH test. IC_50_ values were calculated from the dose–response curves obtained using the 4-PL logistic model (*P* < 0.05). S.D. = standard deviation.

As compound **15** emerged as the most promising derivative from cytotoxicity studies in both 2D and 3D models, it was selected for uptake and mechanistic studies. Derivatives **14** and **16**, bearing the same ligand but a different coligand or a different Cu oxidation state, were included in these studies for useful comparison.

#### Cellular Uptake

**Figure 5:**
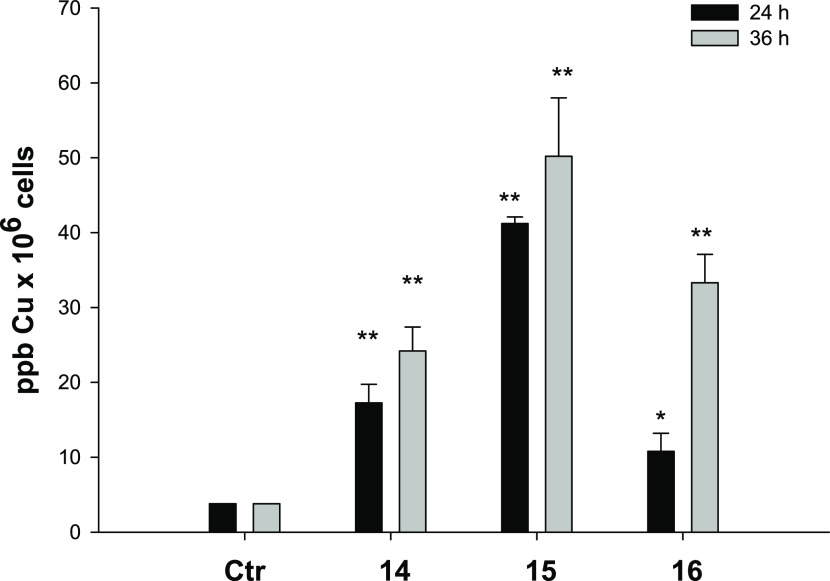
Intracellular copper content after treatment with compounds **14**–**16**. PSN-1 cells were treated for 24 or 36 h with 1 μM copper complexes, and the intracellular copper amount was estimated using GF-AAS analysis. Error bars indicate the standard deviation. *P < 0.1 and **P < 0.01 compared with the control.

As stated before, copper accumulation in cancer cells is one of the most important factors affecting copper complex cytotoxicity. In an attempt to correlate cytotoxic activity with cellular accumulation, copper content was evaluated in PSN-1 cells treated for 24 or 36 h with 1 μM of complexes **14**–**16**. The intracellular copper amount was quantified by means of graphite furnace atomic absorption spectrometry (GF-AAS) analysis, and the results, expressed as metal parts per billion per 10^6^ cells, are shown in Figure 5.

Although to a different extent, all three copper complexes accumulated into cancer cells. Notably, the intracellular Cu levels follow the trend **15** > **14** > **16** after 24 h, thus suggesting that Cu(I) complexes are more effective in crossing the cancer cell membrane with respect to the Cu(II) derivative. Interestingly, in the case of the Cu(II) complex **16**, the intracellular copper levels significantly increased with exposure time, whereas Cu(I) complexes **14** and **15** seemed to accumulate in a time-independent manner. These results might suggest the involvement of different internalization mechanisms for Cu(I) and Cu(II) complexes, whose electronic and molecular structures have been confirmed using XPS and XAS analysis. Moreover, by comparing uptake and cytotoxicity data in human pancreatic PSN-1 cancer cells, a direct correlation between cellular accumulation and cytotoxic potency can be highlighted.

### Mechanistic Studies

Copper complexes have been regarded as redox active modulators as they may catalyze hydrogen peroxide in the form of Fenton-like reactions inside the cell to produce reactive oxygen species (ROS), thus altering the cellular redox homeostasis and driving cells toward oxidative stress.^76^ In addition, it has previously been reported that LND acts as an antitumor drug by inhibiting both mitochondrial respiration and glycolysis, thus shifting cultured cells to a more oxidized redox state.^77^ Moreover, some classes of copper complexes were found to exert an effective antiproliferative action by dysregulating the mitochondrial function in cancer cells.^47^

**Figure 6:**
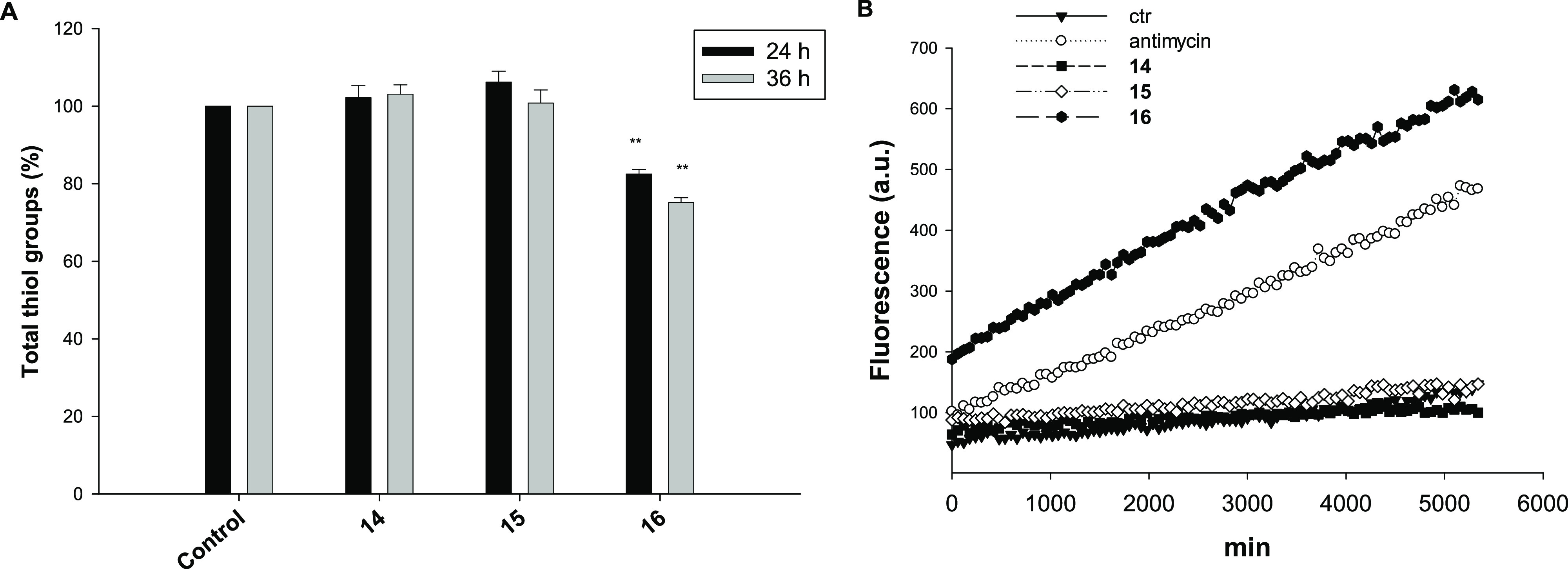
(A) Sulfhydryl content in PSN1-treated cancer cells incubated for 24 or 36 h with tested compounds **14**–**16**. The sulfhydryl group amount was determined using the DTNB assay. Error bars indicate the S.D. **P < 0.01 compared with the control. (B) Effect of copper compounds on hydrogen peroxide formation in PSN-1 cells. PSN-1 cells were preincubated in PBS/10 mM glucose medium for 20 min at 37 °C in the presence of 10 μM CM-DCFDA and then treated with 10 μM of tested compounds.

On these bases, we evaluated the ability of **14**–**16** to alter cellular redox homeostasis, in terms of total cellular sulfhydryl content, ROS production, and perturbation of the mitochondrial membrane potential in PSN-1 cells (Figure 6). For compounds **15** and **16**, SR-XPS data analysis provided information about the electronic and molecular structures, evidencing the molecular structural stability for both Cu(I) and Cu(II) complexes and confirming the expected oxidation states.

Interestingly, Cu(I) complexes **14** and **15** were completely ineffective in modulating the total thiol content in PSN1-treated cancer cells, whereas treatment with **16** induced a substantial time-dependent alteration of the total cellular sulfhydryl content, determining 18 and 25% reduction of thiol groups with respect to control untreated cells following 24 or 36 h of exposure, respectively (Figure 6A).

Consistently, treatment of PSN-1 cells with complex **16** determined a substantial time-dependent increase in cellular basal ROS production, whereas treatment with **14** and **15** did not result in an increase in the basal cellular ROS production (Figure 6B). Notably, treatment with **16** determined a substantial increase in basal hydrogen peroxide formation, which was even higher than that obtained with antimycin, a classical inhibitor of the mitochondrial respiratory chain at the level of complex III.

Overall, these results demonstrate that the newly developed Cu(II) complex **16** induced an oxidative shift in the redox status of PSN-1 cells. On the contrary, Cu(I) complexes **14** and **15** seemed to act through a mechanism of action that does not encompass oxidative stress induction.

A persistent increase in the rate of ROS production and the induction of thiol redox stress can in turn prompt the collapse of the mitochondrial membrane potential as well as the loss of the mitochondrial shape and integrity (*swelling*), possibly leading to the induction of cell apoptosis.^78^ We hence evaluated the effect determined by treatment with complexes **14**–**16** in terms of the modification of mitochondrial pathophysiological characteristics, such as the mitochondrial membrane potential and induction of cell death.

**Figure 7:**
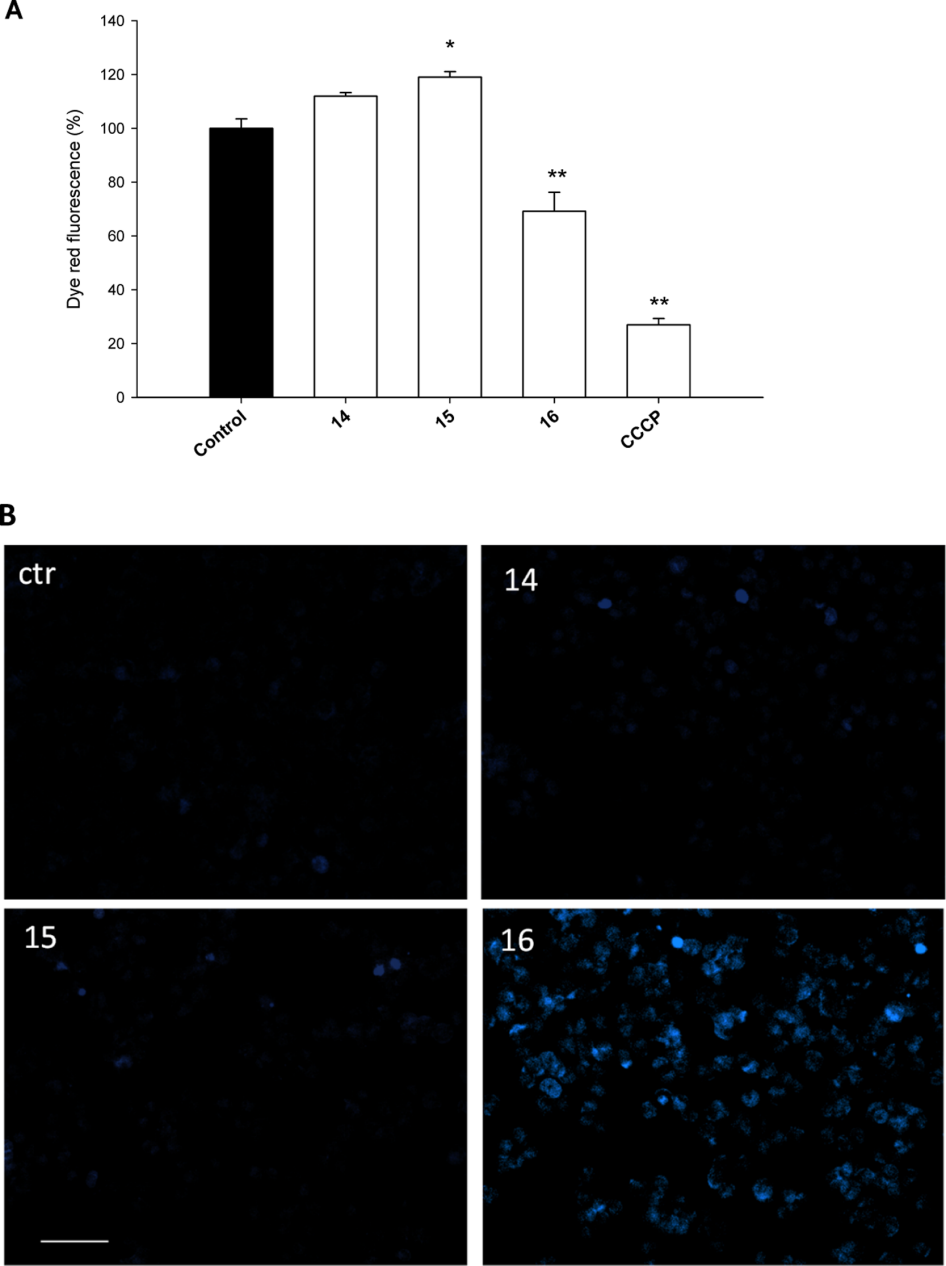
(A) PSN-1 cells were treated for 24 h with IC_50_ concentrations of tested complexes or CCCP (3 μM). The mitochondrial membrane potential was determined using the Mito-ID membrane potential kit. Data are the means of three independent experiments. Error bars indicate the S.D. *P < 0.1 and **P < 0.01 compared with the control. (B) Hoechst staining of PSN-1 cells incubated for 48 h with IC_50_ doses of **14**, **15**, or **16**.

For mitochondrial membrane potential detection, PSN-1 cells were treated with IC_50_ concentrations of the tested complexes, and the percentage of cells with the altered mitochondrial membrane potential was determined by means of the Mito-ID membrane potential kit. As evident by results depicted in Figure 7A, as expected, complex **16** induced a 31% decrease in the dye red fluorescence, rather similar to that induced by the reference compound carbonyl cyanide-*m*-chlorophenylhydrazone (CCCP), thus attesting a significant increase in the percentage of hypopolarized cells. On the other hand, **14** and **15** induced a slight (about 12 and 19%, respectively) increase in the dye red fluorescence, thus indicating a modest increase in cancer cell population with the hyperpolarized mitochondrial membrane potential.

Considering these results, it is possible to state that Cu(I) complexes **14** and **15** possess a rather different mechanism of action compared to Cu(II) complex **16**.

It has been widely described that Cu(I) and Cu(II) complexes can induce different types of cell death. In order to analyze the mechanism involved in the loss of cancer cell viability, we assessed the ability of selected complexes to induce cancer cell death by means of apoptosis. Figure 7B shows the results obtained upon monitoring the cellular morphological changes in PSN-1 cells treated for 48 h with IC_50_ doses of **14**, **15**, and **16** and stained with a Hoechst 33258 fluorescent probe. Compared with control cells, cells treated with **16** presented brightly stained nuclei and morphological features typical of cells undergoing apoptosis, such as chromatin condensation and fragmentation, thus confirming the ability of Cu(II) complex **16** to induce cancer cell death by means of apoptosis. Conversely, cells treated with **14** and **15** did not show any classical sign of apoptosis induction and appeared increased in size and ultrastructural complexity.

These data are consistent with those already obtained with other series of similarly unconjugated or differently conjugated (pyrazolyl)acetate complexes and once again point out to the importance of the development of Cu(I) species able to trigger paraptosis, a strategic approach toward cancer cells that have become resistant to the apoptosis inducer drugs.^49,52,73,74^

Altogether, these results clearly suggest that Cu(II) complex **16** triggers cancer cell death via an apoptotic pathway, whereas Cu(I) complexes **14** and **15** kill cancer cells by means of an apoptosis alternative cancer cell death, possibly paraptosis, that has been recognized as a strategic pathway in cancer cells that are resistant to apoptotic mechanisms. Paraptosis is a type of programmed cell death, morphologically distinct from apoptosis and necrosis. Defining features of paraptosis are cytoplasmic vacuolation and the lack of an apoptotic morphology (cell shrinkage, apoptotic bodies, chromatin condensation, and nuclear fragmentation). Like apoptosis and other types of programmed cell death, the cell is involved in causing its own death, and gene expression is required.^79^

Interestingly, the above discussed EXAFS data analysis revealed that in Cu(I) complex **15**, the Cu–N nearest neighbor distance is slightly longer with respect to Cu(II) complexes **10** and **16**, suggesting more loosely bound copper in complex **15**, likely related to the lower electronegativity of Cu(I). This structural feature, potentially leading to a faster ligand exchange rate and reactivity, might be responsible for the higher antitumor efficacy of **15**.

Considering all the collected data, complex **15** can be recommended for a more detailed investigation of its biological properties, such as the assessment of its interactions with important biomolecules and the evaluation of the in vivo efficacy.

## Conclusions

In this study, the known antitumor drug LND was functionalized with species able to coordinate metals, affording new heteroscorpionate ligands **1**–**4**, which were used for the preparation of Cu(I) and Cu(II) complexes **5**–**16**. In the solid state, a multitechnique approach (SR-XPS, NEXAFS, and XAFS) allowed us to ascertain the molecular stability of the ligands upon interaction with the copper ions, as well as to determine the coordination geometry and copper ion oxidation state.

From the biological studies, the following promising results emerged:

i All the complexes showed an extremely promising cytotoxic activity in a panel of human tumor cell lines, eliciting IC_50_ values in the low or submicromolar range and being more effective than the reference metallodrug cisplatin.
ii Noteworthily, they were able to overcome the oxaliplatin and multidrug resistance.
iii They were also more effective than cisplatin against the more predictive 3D spheroids of pancreatic PSN-1 cancer cells. In particular, the lipophilic PPh_3_-containing Cu(I) complexes proved to be the most active compounds, with complex **15** being the most promising candidate.
iv Representative complexes **14**–**16** were able to cross the cellular plasmalemma, and Cu accumulated differently in treated cancer cells with a direct correlation between cellular accumulation and cytotoxic potency, suggesting the involvement of different internalization mechanisms for Cu(I) and Cu(II) complexes.
v More importantly, Cu(I) complexes proved to be ineffective in modulating cellular oxidative stress and induced cancer cell death via an apoptosis-alternative pathway, possibly paraptosis. On the contrary, Cu(II) complexes caused oxidative stress and triggered apoptotic cell death.

Based on their interesting biological profile, Cu(I) LND-conjugated complexes deserve to be further investigated, with the aim to find application in solid tumors, refractory to platinum-based drug treatment.

## Experimental Section

### Chemistry

#### Materials and General Methods

All the reagents have been purchased and used without further purification. Melting points (mps) were performed using an SMP3 Stuart Scientific Instrument (Bibby Sterilin Ltd., London, UK). Elemental analyses (C, H, N, and S) (EA) were performed using a Fisons Instruments EA-1108 CHNS–O elemental analyzer (Thermo Fisher Scientific Inc., Waltham, MA, USA). Fourier transform infrared (FT-IR) spectra were recorded from 4000 to 700 cm^–1^ on a PerkinElmer Frontier instrument (PerkinElmer Inc., Waltham, MA, USA), equipped with an attenuated total reflection unit using a universal diamond top plate as a sample holder. Abbreviation used in the analyses of the FT-IR spectra: br = broad, m = medium, mbr = medium broad, s = strong, sbr = strong broad, sh = shoulder, vs = very strong, w = weak, and wbr = weak broad. NMR spectra for nuclei ^1^H, ^13^C, and ^31^P were recorded using a Bruker 500 Ascend spectrometer (Bruker BioSpin Corporation, Billerica, MA, USA; 500.1 MHz for ^1^H, 125 MHz for ^13^C, and 202.4 MHz for ^31^P). Tetramethylsilane [Si(CH_3_)_4_] was used as an external standard for the ^1^H and ^13^C NMR spectra, while 85% H_3_PO_4_ was used for the ^31^P NMR spectra. The chemical shifts (δ) are reported in parts per million. Abbreviation used in the analyses of the NMR spectra: br = broad, d = doublet, m = multiplet, s = singlet, sbr = singlet broad, t = triplet, and tbr = triplet broad. ESI-MS spectra were recorded in the positive- [ESI-MS(+)] or negative-ion [ESI-MS(−)] mode on a Waters Micromass ZQ spectrometer equipped with a single quadrupole (Waters Corporation, Milford, MA, USA), using a methanol or acetonitrile mobile phase. The compounds were added to reagent grade methanol or acetonitrile to give approximately 0.1 mM solutions. These solutions were injected (1 μL) into the spectrometer fitted with an autosampler. The pump delivered the solutions to the mass spectrometer source at a flow rate of 200 μL/min, and nitrogen was employed both as a drying gas and as a nebulizing gas. Capillary voltage was typically 2500 V. The temperature of the source was 100 °C, while the temperature of the desolvation was 400 °C. In the analyses of ESI-MS spectra, the confirmation of major peaks was supported by comparison of the observed and predicted isotope distribution patterns, the latter calculated using IsoPro 3.1 computer software (T-Tech Inc., Norcross, GA, USA).

#### Synthesis of 2-Hydroxyethyl-1-(2,4-dichlorobenzyl)-1*H*-indazole-3-carboxylate (LONES)

Sulfuric acid (2 mL) was added to a solution of LND (0.50 g, 1.56 mmol) in ethylene glycol (35 mL), and the reaction mixture was stirred at 85 °C for 1 h. After cooling to room temperature, the solution was poured into the ice water. The aqueous layer (50 mL) was extracted by dichloromethane (3 × 30 mL). The organic phase was dried over Na_2_SO_4_. The evaporation of the solvent under reduced pressure afforded a residue, which was purified using column chromatography, eluting with cyclohexane/EtOAc (6:4). A white solid was obtained (65% yield). mp: 121–122 °C. IR (cm^–1^): 3480mbr (O–H); 3084wbr, 2949wbr (C–H); 1713s (C=O); 1585m; 1560sh (C=C/C=N); 1479m, 1472s, 1446m, 1436s, 1421m, 1311s, 1275m, 1249s, 1225s, 1167s, 1157s, 1129s, 1096m, 1084s, 1044m, 1025m, 1009m, 990m, 977m, 944m, 898m, 855m, 839m, 807m, 789m, 776m, 747s, 737s. ^1^H NMR (CDCl_3_, 293 K): δ 4.07 (m, 2H, −C*H*_2_–OH), 4.65 (m, 2H, −C*H*_2_–O), 5.81 (s, 2H, N–C*H*_2_-Ph), 6.72 (d, 1H, Ar*H*), 7.10–7.49 (m, 6H, Ar*H* and O*H*), 8.28 (d, 1H, Ar*H*). ESI-MS (major positive ions, CH_3_OH), *m*/*z* (%): 365 (20) [LONES + H]^+^, 387 (100) [LONES + Na]^+^, 753 (60) [2LONES + Na]^+^. ESI-MS (major negative ions, CH_3_OH), *m*/*z* (%): 363 (100) [LONES – H]^−^.

#### Synthesis of *N*-(2-Aminoethyl)-1-(2,4-dichlorobenzyl)-1*H*-indazole-3-carboxamide (LONAM)

CDI (0.61 g, 3.74 mmol) was added to a solution of LND (1.00 g, 3.12 mmol) in anhydrous tetrahydrofuran (THF) (20 mL), and the mixture was stirred at room temperature for 1 h. After ethylenediamine (0.94 g, 15.6 mmol) was added, the solution was stirred at room temperature for 18 h. After evaporation of the solvent, the oil formed was dissolved in CHCl_3_ (30 mL) and washed with H_2_O (2 × 20 mL). The organic phase was dried over Na_2_SO_4_. The evaporation of the solvent under reduced pressure afforded a residue, which was purified using column chromatography, eluting with MeOH/EtOAc (1:9). A white solid was obtained (65% yield). mp: 71–72 °C. IR (cm^–1^): 3282br (N–H); 3060w, 3030w, 2937wbr (C–H); 1657sh, 1642s (C=O); 1620sh, 1588m; 1575m, 1538s (C=C/C=N); 1491s, 1473s, 1436s, 1406m, 1386m, 1372m, 1357m, 1310sbr, 1282m, 1233s, 1175s, 1155m, 1132m, 1097s, 1048s, 1005m, 968mbr, 942m, 859m, 836s, 788s, 772s, 742s. ^1^H NMR (CDCl_3_, 293 K): δ 3.01–3.58 (m, 4H, NH–C*H*_2_–C*H*_2_–NH), 4.87 (br, 2H, N*H*_2_), 5.64 (s, 2H, N–C*H*_2_–Ph), 6.63 (d, 1H, Ar*H*), 7.02–7.48 (m, 6H, Ar*H* and N*H*), 8.46 (d, 1H, Ar*H*). ESI-MS (major positive ions, CH_3_OH), *m*/*z* (%): 363 (100) [LONAM + H]^+^, 385 (20) [LONAM + Na]^+^, 727 (40) [2LONAM + H]^+^. ESI-MS (major negative ions, CH_3_OH), *m*/*z* (%): 361 (100) [LONAM – H]^−^.

#### Synthesis of L^1^ (**1**)

A solution of HC(pz)_2_COOH (0.300 g, 1.560 mmol), LONES (0.624 g, 1.710 mmol), and DMAP (0.020 g, 0.157 mmol) in THF (20 mL) was cooled to 0 °C. EDCI·HCl (0.360 g, 1.880 mmol) was added, and the mixture was stirred for 16 h at room temperature. The reaction was then quenched with H_2_O (30 mL), and the aqueous phase was extracted with EtOAc (2 × 30 mL) The organic phase was washed with brine (2 × 20 mL) and dried over Na_2_SO_4_. The evaporation of the solvent afforded a residue, which was purified using column chromatography, eluting with cyclohexane/EtOAc (7:3). A white solid was obtained (72% yield). mp: 95–96 °C. IR (cm^–1^): 3121wbr, 2958wbr (C–H); 1762m, 1716mbr (C=O); 1616w; 1590w, 1564w, 1518w (C=C/C=N); 1476m, 1434m, 1387s, 1317m, 1291m, 1259m, 1221s, 1191m, 1156s, 1123s, 1088s, 1046s, 1008m, 967m, 948m, 916m, 884m, 835m, 817m, 801m, 788m, 748s. ^1^H NMR (DMSO-*d*_6_, 293 K): δ 4.52–4.61 (m, 4H, O–C*H*_2_–C*H*_2_–O), 5.89 (s, 2H, N–C*H*_2_–Ph), 6.26 (t, 2H, 4-C*H*), 6.96 (d, 1H, Ar*H*), 7.37–8.01 (m, 11H, 3-C*H*, 5-C*H*, Ar*H* and C*H*CO). ^1^H NMR (CDCl_3_, 293 K): δ 4.72 (m, 4H, O–C*H*_2_–C*H*_2_–O), 5.81 (s, 2H, N–C*H*_2_-Ph), 6.30 (m, 2H, 4-C*H*), 6.73 (d, 1H, Ar*H*), 7.11–7.81 (m, 10H, 3-C*H*, 5-C*H*, Ar*H* and C*H*CO), 8.15 (d, 1H, Ar*H*). ESI-MS (major positive ions, CH_3_OH) *m*/*z* (%): 539 (20) [L^1^ + H]^+^, 561 (100) [L^1^ + Na]^+^. Calcd for C_25_H_20_Cl_2_N_6_O_4_: H, 3.74; C, 55.67; N, 15.58%. Found: H, 3.94; C, 55.33; N, 15.20%.

#### Synthesis of L^2^ (**2**)

This compound was prepared from HC(3,5-Me_2_pz)_2_COOH and LONES following the procedure described for L^1^ (**1**): a white solid was obtained (75% yield). mp: 139–140 °C. IR (cm^–1^): 3063wbr, 2958w, 2928w (C–H); 1763s, 1726sbr (C=O); 1616w; 1590m, 1562s (C=C/C=N); 1476s, 1416s, 1378s, 1316s, 1265s, 1218vs, 1202vs, 1156vs, 1123vs, 1102vs, 1048s, 1035vs, 1008s, 970s, 947m, 892m, 862m, 835s, 787vs, 748vs. ^1^H NMR (DMSO-*d*_6_, 293 K): δ 1.96 (s, 6H, C*H*_3_), 2.09 (s, 6H, C*H*_3_), 4.55–4.63 (m, 4H, O–C*H*_2_–C*H*_2_–O), 5.79 (s, 2H, N–C*H*_2_-Ph), 5.88 (s, 2H, 4-C*H*), 6.97 (d, 1H, Ar*H*), 7.33–8.05 (m, 7H, Ar*H* and C*H*CO). ^1^H NMR (CDCl_3_, 293 K): δ 2.11 (s, 6H, C*H*_3_), 2.14 (s, 6H, C*H*_3_), 4.74 (m, 4H, O–C*H*_2_–C*H*_2_–O), 5.81, (s, 2H, N–C*H*_2_-Ph), 5.83 (s, 2H, 4-C*H*), 6.73 (d, 1H, Ar*H*), 7.06–8.16 (m, 7H, Ar*H* and C*H*CO). ESI-MS (major positive ions, CH_3_OH), *m*/*z* (%): 595 (20) [L^2^ + H]^+^, 617 (100) [L^2^ + Na]^+^, 633 (20) [L^2^ + K]^+^, 1213 (10) [2L^2^ + Na]^+^. Calcd for C_29_H_28_Cl_2_N_6_O_4_: H, 4.74; C, 58.49; N, 14.11%. Found: H, 4.45; C, 58.71; N, 13.88%.

#### Synthesis of L^3^ (**3**)

A mixture of HC(pz)_2_COOH (0.156 g, 0.810 mmol), LONAM (0.330 g, 0.900 mmol), EDCI·HCl (0.230 g, 1.20 mmol), HOBt (0.160 g, 1.18 mmol), and triethylamine (0.107 mg, 1.06 mmol) in *N*,*N*-dimethylformamide (DMF) (10 mL) was stirred at room temperature for 16 h. The reaction was quenched with H_2_O (30 mL), and the aqueous phase was extracted with EtOAc (2 × 20 mL). The organic phase was washed with brine (3 × 20 mL) and NaHCO_3_ (3 × 20 mL) and dried over Na_2_SO_4_. The evaporation of the solvent afforded a residue, which was purified using column chromatography, eluting with cyclohexane/EtOAc (3:7). A white solid was obtained (68% yield). mp: 179–181 °C. IR (cm^–1^): 3275mbr, 3223m (N–H); 3075m, 2951m, (C–H); 1673vs, 1641vs (C=O); 1587m, 1539vs, 1515s (C=C/C=N); 1493s, 1473s, 1449s, 1433m, 1389vs, 1371s, 1313s, 1285s, 1249s, 1230vs, 1195s, 1177vs, 1154s, 1131m, 1112m, 1099s, 1092s, 1084s, 1064m, 1049vs, 1005m, 985w, 969m, 946m, 935m, 915m, 896w, 883w, 864s, 837vs, 812vs, 788s, 751vs. ^1^H NMR (DMSO-*d*_6_, 293 K): δ 3.30–3.47 (m, 4H, NH–C*H*_2_–C*H*_2_–NH), 5.83 (s, 2H, N–C*H*_2_-Ph), 6.27 (t, 2H, 4-C*H*), 6.78 (d, 1H, Ar*H*), 7.25–8.64 (m, 13H, 3-C*H*, 5-C*H*, Ar*H*, C*H*CO and N*H*). ^1^H NMR (CDCl_3_, 293 K): δ 3.69 (m, 4H, NH–C*H*_2_–C*H*_2_–NH), 5.67 (s, 2H, N–C*H*_2_-Ph), 6.26 (m, 2H, 4-C*H*), 6.68 (d 1H, Ar*H*), 7.00–7.72 (m, 12H, 3-C*H*, 5-C*H*, Ar*H*, C*H*CO and N*H*), 8.38 (d, 1H, Ar*H*). ESI-MS (major positive ions, CH_3_OH), *m*/*z* (%): 559 (100) [L^3^ + Na]^+^, 1097 (30) [2L^3^ + Na]^+^. ESI-MS (major negative ions, CH_3_OH), *m*/*z* (%): 557 (100) [L^3^ – H]^−^. Calcd for C_25_H_22_Cl_2_N_8_O_2_: H, 4.13; C, 55.87; N 20.85%. Found: H, 3.97; C, 55.53; N, 20.59%.

#### Synthesis of L^4^ (**4**)

This compound was prepared from HC(3,5-Me_2_pz)_2_COOH and LONAM following the procedure described for L^3^ (**3**): a white solid was obtained (75% yield). mp: 170–172 °C. IR (cm^–1^): 3288br (N–H); 3070wbr, 2947wbr (C–H); 1669s, 1644s (C=O); 1590m, 1562sh, 1536s (C=C/C=N); 1491m, 1473s, 1456m, 1439s, 1415m, 1374s, 1363m, 1338m, 1312s, 1272s, 1246s, 1228s, 1198m, 1178s, 1154m, 1111m, 1099m, 1081m, 1050m, 1033m, 1028m, 1002m, 974m, 948m, 885m, 861m, 834s, 807s, 794m, 780s, 751vs, 740s, 709s. ^1^H NMR (DMSO-*d*_6_, 293 K): δ 2.02 (s, 6H, C*H*_3_), 2.08 (s, 6H, C*H*_3_), 3.37–3.47 (m, 4H, NH–C*H*_2_–C*H*_2_–NH), 5.81 (s, 2H, N–C*H*_2_-Ph), 5.83 (s, 2H, 4-C*H*), 6.74 (d, 1H, Ar*H*), 6.91–8.38 (m, 9H, Ar*H*, C*H*CO and N*H*). ^1^H NMR (CDCl_3_, 293 K): δ 2.09 (s, 6H, C*H*_3_), 2.30 (s, 6H, C*H*_3_), 3.62–3.76 (m, 4H, NH–C*H*_2_–C*H*_2_–NH), 5.66 (s, 2H, N–C*H*_2_-Ph), 5.80 (s, 2H, 4-C*H*), 6.67 (d, 1H, Ar*H*), 6.97–8.36 (m, 8H, Ar*H*, C*H*CO and N*H*). ESI-MS (major positive ions, CH_3_OH), *m*/*z* (%): 593 (100) [L^4^ + H]^+^, 615 (50) [L^4^ + Na]^+^. ESI-MS (major negative ions, CH_3_OH), *m*/*z* (%): 591 (100) [L^4^ – H]^−^. Calcd for C_29_H_30_Cl_2_N_8_O_2_: H, 5.10; C, 58.69; N, 18.88%. Found: H, 4.97; C, 58.43; N, 19.14%.

#### Synthesis of [(PTA)_2_Cu(L^1^)]PF_6_ (**5**)

PTA (0.117 g, 0.743 mmol) was added to a solution of Cu(CH_3_CN)_4_PF_6_ (0.139 g, 0.372 mmol) in acetonitrile (80 mL). The reaction mixture was stirred at room temperature for 4 h; then, L^1^ (**1**, 0.200 g, 0.372 mmol) was added, and the suspension was stirred overnight. The reaction mixture was filtered and dried under reduced pressure to give the crystalline white complex [(PTA)_2_Cu(L^1^)]PF_6_ (**5**) in 73% yield. mp: 147–150 °C. IR (cm^–1^): 3128wbr, 2937wbr (C–H); 1761m, 1718m (C=O); 1616w; 1590w, 1564w, 1519w (C=C/C=N); 1477m, 1446m, 1418m, 1403m, 1390m, 1289m, 1242m, 1218m, 1161m, 1126m, 1098m, 1049m, 1015m, 971s, 948m, 917w, 893w, 876w, 833vs, 817m, 789m, 752s, 742s. ^1^H NMR (DMSO-*d*_6_, 293 K): δ 4.07 (sbr, 12H, NC*H*_2_P), 4.41–4.65 (m, 16H, NC*H*_2_N and OC*H*_2_C*H*_2_O), 5.89 (s, 2H, NC*H*_2_Ph), 6.35 (sbr, 2H, 4-C*H*), 6.98 (d, 1H, Ar*H*), 7.39–8.02 (m, 11H, 3-C*H*, 5-C*H*, C*H*CO and Ar*H*). ^13^C NMR (DMSO-*d*_6_, 293 K): δ 49.7 (N*C*H_2_Ph); 50.2, 50.9 (*C*H_2_P); 64.2, 66.0 (O*C*H_2_*C*H_2_O); 71.4 (*C*H_2_N); 106.2 (4-*C*H); 110.4, 121.3, 123.1, 123.2, 127.0, 127.5, 128.8, 130.6, 130.7, 132.8, 140.6, 141.5 (ArH, Ar, *C*HCO, 3-*C*H and 5-*C*H); 160.8, 163.8 (*C*O). ^31^P{^1^H} NMR (DMSO-*d*_6_, 293 K): δ −144.18 (septet, *J*(F–P) = 711 Hz, PF_6_), −95.02 (br). ^31^P{^1^H} NMR (CD_3_CN, 293 K): δ −143.49 (septet, *J*(F–P) = 706 Hz, PF_6_), −90.42 (br). ESI-MS (major positive ions, CH_3_CN), *m*/*z* (%): 158 (20) [PTA + H]^+^, 603 (100) [Cu(L^1^)]^+^. ESI-MS (major negative ions, CH_3_CN), *m*/*z* (%): 145 (100) [PF_6_]^−^. Calcd for C_37_H_44_Cl_2_CuF_6_N_12_O_4_P_3_: H, 4.18; C, 41.84; N, 15.82%. Found: H, 4.12; C, 41.67; N, 15.47%.

#### Synthesis of [(PPh_3_)_2_Cu(L^1^)]PF_6_ (**6**)

This compound was prepared from PPh_3_ following the procedure described for **5**. The obtained residue was filtered and dried under reduced pressure to give the crystalline white complex [(PPh_3_)_2_Cu(L^1^)]PF_6_ (**6**) in 74% yield. mp: 105–109 °C. IR (cm^–1^): 3133wbr, 3056wbr (C–H); 1765m, 1716mbr (C=O); 1616w; 1588m, 1564w, 1523w (C=C/C=N); 1479m, 1455m, 1435m, 1403m, 1353m, 1299m, 1286m, 1216m, 1161s, 1125m, 1095s, 1055m, 1027m, 1008m, 999m, 986m, 955m, 919w, 834vs, 788m, 742vs. ^1^H NMR (DMSO-*d*_6_, 293 K): δ 4.47 (sbr, 4H, OC*H*_2_C*H*_2_O), 5.88 (s, 2H, NC*H*_2_Ph), 6.28 (sbr, 2H, 4-C*H*), 6.98 (d, 1H, Ar*H*), 7.26–7.99 (m, 41H, 3-C*H*, 5-C*H*, C*H*CO and Ar*H*). ^13^C NMR (DMSO-*d*_6_, 293 K): δ 52.7 (N*C*H_2_Ph); 64.7, 67.2 (O*C*H_2_*C*H_2_O); 106.1 (4-*C*H); 110.6, 122.4, 122.6, 123.0, 128.6, 128.8, 129.1, 129.3, 129.4, 130.0, 131.7, 132.5, 132.6, 132.8, 133.0, 133.4, 133.8, 138.8, 142.4 (ArH, Ar, *C*HCO, 3-*C*H and 5-*C*H); 161.6, 163.9 (*C*O). ^31^P{^1^H} NMR (DMSO-*d*_6_, 293 K): δ −148.80 (septet, *J*(F–P) = 713 Hz, PF_6_), −3.33 (br). ^31^P{^1^H} NMR (CD_3_CN, 293 K): δ −143.51 (septet, *J*(F–P) = 705 Hz, PF_6_), 0.15 (br). ESI-MS (major positive ions, CH_3_CN), *m*/*z* (%): 587 (100) [Cu(PPh_3_)_2_]^+^, 603 (40) [Cu(L^1^)]^+^. ESI-MS (major negative ions, CH_3_CN), *m*/*z* (%): 145 (100) [PF_6_]^−^. Calcd for C_61_H_50_Cl_2_CuF_6_N_6_O_4_P_3_: H, 3.96; C, 57.58; N, 6.60%; Found: H, 3.98; C, 57.27; N, 6.39%.

#### Synthesis of [(L^1^)CuCl_2_] (**7**)

CuCl_2_·2H_2_O (0.063 g, 0.371 mmol) was added to an acetonitrile solution (80 mL) of L^1^ (**1**, 0.200 g, 0.371 mmol). The reaction mixture was stirred at room temperature for 24 h to obtain a precipitate, which was filtered, washed with acetonitrile, and dried under reduced pressure to give the green complex [(L^1^)CuCl_2_] (**7**) in 87% yield. mp: 193–195 °C. IR (cm^–1^): 3129w, 3112wbr, 2996w (C–H); 1748s, 1710s (C=O); 1591w, 1567w (C=C/C=N); 1500w, 1478m, 1452m, 1429m, 1403s, 1361w, 1301m, 1284m, 1254m, 1230m, 1220s, 1195m, 1168vs, 1129s, 1090s, 1060s, 1040m, 1008m, 994m, 953m, 920w, 891m, 852m, 818m, 800m, 781s, 764s, 751s. ESI-MS (major positive ions, CH_3_CN), *m*/*z* (%): 602 (100) [Cu(L^1^) – H]^+^. ESI-MS (major negative ions, CH_3_CN), *m*/*z* (%): 170 (100) [CuCl_3_]^−^. Calcd for C_25_H_20_Cl_4_CuN_6_O_4_: H, 2.99; C, 44.56; N, 12.47%. Found: H, 3.11; C, 44.80; N, 12.71%.

#### Synthesis of [(PTA)_2_Cu(L^3^)]PF_6_ (**8**)

This compound was prepared from L^3^ (**3**) following the procedure described for **5**. The obtained residue was filtered and subsequently purified in diethyl ether (50 mL) and then in *n*-hexane (50 mL) to obtain the crystalline white complex [(PTA)_2_Cu(L^3^)]PF_6_ (**8**) in 57% yield. mp: 163–167 °C. IR (cm^–1^): 3281wbr (N–H); 3127wbr, 2936wbr (C–H); 1691mbr, 1652mbr (C=O); 1589w, 1537mbr (C=C/C=N); 1493m, 1474m, 1446m, 1417sh, 1403m, 1372mbr, 1291m, 1242m, 1177m, 1136w, 1099m, 1048m, 1015s, 970s, 949s, 917w, 894w, 833vs, 742sbr. ^1^H NMR (DMSO-*d*_6_, 293 K): δ 3.39 (s, 4H, NHC*H*_2_C*H*_2_NH), 4.07 (sbr, 12H, NC*H*_2_P), 4.40–4.64 (m, 12H, NC*H*_2_N), 5.83 (s, 2H, NC*H*_2_Ph), 6.42 (br, 2H, 4-C*H*), 6.78 (d, 1H, Ar*H*), 7.32–8.01 (m, 10H, 3-C*H*, 5-C*H*, Ar*H* and C*H*CO), 8.22 (d, 1H, Ar*H*), 8.40 (tbr, 1H, N*H*), 8.46 (sbr, 1H, N*H*). ^13^C NMR (DMSO-*d*_6_, 293 K): δ 38.2 (N*C*H_2_*C*H_2_N); 50.0 (N*C*H_2_Ph); 50.3, 51.8 (*C*H_2_P); 72.3 (*C*H_2_N); 105.5; 107.1 (4-*C*H); 110.8 122.5, 122.7, 123.2, 127.7, 128.3, 129.5, 130.6, 132.4, 133.3, 133.7, 133.9, 138.5, 141.6 (ArH, Ar, *C*HCO, 3-*C*H and 5-*C*H); 162.5, 164.1 (*C*O). ^31^P{^1^H} NMR (DMSO-*d*_6_, 293 K): δ −144.19 (septet, *J*(F–P) = 711 Hz, PF_6_), −93.70 (sbr). ^31^P{^1^H} NMR (CD_3_CN, 293 K): δ −143.51 (septet, *J*(F–P) = 706 Hz, PF_6_), −91.0 (br). ^31^P{^1^H} NMR (CDCl_3_, 293 K): δ −143.80 (septet, *J*(F–P) = 714 Hz, PF_6_), −92.02 (sbr). ESI-MS (major positive ions, CH_3_CN), *m*/*z* (%): 158 (10) [PTA + H]^+^, 220 (5) [Cu(PTA)]^+^, 377 (30) [Cu(PTA)_2_]^+^, 601 (100) [Cu(L^3^)]^+^, 758 (10) [(PTA)Cu(L^3^)]^+^. ESI-MS (major negative ions, CH_3_CN), *m*/*z* (%): 145 (100) [PF_6_]^−^. Calcd for C_37_H_46_Cl_2_CuF_6_N_14_O_2_P_3_: H, 4.37; C, 41.92; N, 18.50%. Found: H, 4.58; C, 41.66; N, 18.18%.

#### Synthesis of [(PPh_3_)_2_Cu(L^3^)]PF_6_ (**9**)

This compound was prepared from PPh_3_ and L^3^ (**3**) following the procedure described for **5**. The obtained residue was filtered and then purified in diethyl ether (50 mL) to obtain the white complex [(PPh_3_)_2_Cu(L^3^)]PF_6_ (**9**) in 65% yield. mp: 186–189 °C. IR (cm^–1^): 3399wbr, 3288wbr (N–H); 3128wbr, 3055wbr (C–H); 1702sh, 1674mbr (C=O); 1626m, 1615m; 1588m, 1540mbr (C=C/C=N); 1479m, 1435s, 1403m, 1373m, 1361m, 1289mbr, 1245mbr, 1231mbr, 1181mbr, 1119m, 1095m, 1056m, 1027m, 999m, 981mbr, 919wbr, 834vs, 741vs. ^1^H NMR (DMSO-*d*_6_, 293 K): δ 3.37–3.41 (m, 4H, NHC*H*_2_C*H*_2_NH), 5.82 (s, 2H, NC*H*_2_Ph), 6.36 (sbr, 2H, 4-C*H*), 6.79 (d, 1H, Ar*H*), 7.31–7.97 (m, 40H, 3-C*H*, 5-C*H*, Ar*H* and C*H*CO), 8.22 (d, 1H, Ar*H*), 8.35 (tbr, 1H, N*H*), 8.65 (tbr, 1H, N*H*). ^13^C NMR (DMSO-*d*_6_, 293 K): δ 38.1 (N*C*H_2_*C*H_2_N); 50.0 (N*C*H_2_Ph); 107.1 (4-*C*H); 110.8, 122.5, 122.7, 123.2, 127.7, 128.3, 129.2, 129.4, 129.5, 130.8, 132.0, 132.5, 132.7, 132.9, 133.4, 133.8, 133.9, 138.5, 141.6, 141.9 (ArH, Ar, *C*HCO, 3-*C*H and 5-*C*H); 162.5, 164.0 (*C*O)·^31^P{^1^H} NMR (DMSO-*d*_6_, 293 K): δ −144.00 (septet, *J*(F–P) = 714 Hz, PF_6_), 0.35 (sbr). ^31^P{^1^H} NMR (CD_3_CN, 293 K): δ −143.53 (septet, *J*(F–P) = 707 Hz, PF_6_), 0.26 (sbr). ESI-MS (major positive ions, CH_3_CN), *m*/*z* (%): 587 (100) [Cu(PPh_3_)_2_]^+^, 601 (10) [Cu(L^3^)]^+^, 863 (20) [(PPh_3_)Cu(L^3^)]^+^. ESI-MS (major negative ions, CH_3_CN), *m*/*z* (%): 145 (100) [PF_6_]^−^. Calcd for C_61_H_52_Cl_2_CuF_6_N_8_O_2_P_3_: H, 4.13; C, 57.67; N, 8.82%. Found: H, 4.06; C, 57.38; N, 8.50%.

#### Synthesis of [(L^3^)CuCl_2_] (**10**)

This compound was prepared from L^3^ (**3**) following the procedure described for **7**, using CH_3_OH as a solvent, to give the light blue complex [(L^3^)CuCl_2_] (**10**) in 68% yield. mp: 216–219 °C dec. IR (cm^–1^): 3494wbr, 3407m, 3290mbr (N–H); 3152w, 3121m, 3091m, 3065w, 2977w, 2943w, 2832w (C–H); 1672vs, 1659sh (C=O); 1565sbr, 1539s (C=C/C=N); 1491s, 1471s, 1450m, 1428m, 1406s, 1384s, 1361m, 1330s, 1313s, 1282vs, 1244s, 1230s, 1205m, 1197m, 1172s, 1150m, 1130m, 1095s, 1063vs, 1046m, 1026m, 1007s, 990s, 956m, 942m, 924m, 892m, 861s, 841s, 833vs, 787vs, 765vs, 748vs, 729s. ESI-MS (major positive ions, CH_3_CN), *m*/*z* (%): 537 (80) [L^3^ + H]^+^, 559 (100) [L^3^ + Na]^+^, 600 (55) [Cu(L^3^) – H]^+^, 1097 (40) [2L^3^ + Na]^+^, 1137 (20) [Cu(L^3^)_2_]^+^. ESI-MS (major negative ions, CH_3_CN), *m*/*z* (%): 170 (10) [CuCl_3_]^−^, 573 (100) [L^3^ + Cl]^−^. Calcd for C_25_H_22_Cl_4_CuN_8_O_2_: H, 3.30; C, 44.69; N, 16.68%. Found: H, 3.52; C, 45.02; N, 16.34%.

#### Synthesis of [(PTA)_2_Cu(L^2^)]PF_6_ (**11**)

This compound was prepared from L^2^ (**2**) following the procedure described for **5**. The reaction was filtered, and the solution was dried under reduced pressure to give the white complex [(PTA)_2_Cu(L^2^)]PF_6_ (**11**) in 94% yield. mp: 187–191 °C. IR (cm^–1^): 3080br, 2985sh, 2950sh, 2925wbr, 2893sh (C–H); 1763m, 1719mbr (C=O); 1647wbr, 1616wbr; 1590w, 1563m (C=C/C=N); 1475m, 1449m, 1418m, 1389m, 1316m, 1297mbr, 1268mbr, 1242m, 1218mbr, 1163mbr, 1123m, 1104m, 1043mbr, 1014s, 969sbr, 948s, 895m, 874sh, 834vs, 803sh, 744s, 718m. ^1^H NMR (DMSO-*d*_6_, 293 K): δ 2.05 (s, 6H, C*H*_3_), 2.36 (s, 6H, C*H*_3_), 4.04 (s, 12H, NC*H*_2_P), 4.40–4.73 (m, 16H, NC*H*_2_N and OC*H*_2_C*H*_2_O), 5.79–5.96 (m, 4H, NC*H*_2_Ph and 4-C*H*), 6.90–8.03 (m, 8H, C*H*CO and Ar*H*). ^13^C NMR (DMSO-*d*_6_, 293 K): δ 11.0 (*C*H_3_); 14.3 (*C*H_3_); 50.3 (N*C*H_2_Ph); 50.5, 51.8 (*C*H_2_P); 72.4 (*C*H_2_N); 65.1, 66.7 (O*C*H_2_*C*H_2_O); 106.2 (4-*C*H); 111.3, 122.0, 123.2, 123.9, 127.9, 128.2, 129.6, 131.6, 133.5, 135.0, 141.4, 144.3, 148.2, 151.4 (ArH, Ar, *C*HCO, 3-*C*H and 5-*C*H); 161.9, 163.4 (*C*O). ^31^P{^1^H} NMR (DMSO-*d*_6_, 293 K): δ −144.19 (septet, *J*(F–P) = 711 Hz, PF_6_), −92.78 (sbr). ^31^P{^1^H} NMR (CD_3_CN, 293 K): δ −143.52 (septet, *J*(F–P) = 706 Hz, PF_6_), −94.0 (br). ESI-MS (major positive ions, CH_3_CN), *m*/*z* (%): 659 (100) [Cu(L^2^)]^+^. ESI-MS (major negative ions, CH_3_CN), *m*/*z* (%): 145 (100) [PF_6_]^−^. Calcd for C_41_H_52_Cl_2_CuF_6_N_12_O_4_P_3_: H, 4.69; C, 44.03; N, 15.03%. Found: H, 4.80; C, 43.77; N, 14.78%.

#### Synthesis of [(PPh_3_)_2_Cu(L^2^)]PF_6_ (**12**)

This compound was prepared from PPh_3_ and L^2^ (**2**) following the procedure described for **5**. The reaction was filtered, and the solution was dried under reduced pressure. Then, the residue in the round-bottom flask was purified with hot diethyl ether (3 × 30 mL) to give the white complex [(PPh_3_)_2_Cu(L^2^)]PF_6_ (**12**) in 51% yield. mp: 118–122 °C. IR (cm^–1^): 3055wbr, 2959wbr, 2924wbr (C–H); 1763m, 1718mbr (C=O); 1635w, 1616w; 1588w, 1563m (C=C/C=N); 1478m, 1435m, 1422sh, 1389m, 1315m, 1265mbr, 1217mbr, 1163m, 1123m, 1095m, 1049m, 1029m, 1008w, 998m, 961w, 896w, 875m, 837vs, 789m, 743s. ^1^H NMR (DMSO-*d*_6_, 293 K): δ 1.92 (s, 6H, C*H*_3_), 2.41 (s, 6H, C*H*_3_), 3.70, 4.27 (sbr, 4H, OC*H*_2_C*H*_2_O), 5.88 (s, 2H, NC*H*_2_Ph), 6.02 (s, 2H, 4-C*H*), 7.07 (d, 1H, Ar*H*), 7.28–7.71 (m, 35H, C*H*CO and Ar*H*), 7.93 (d, 1H, Ar*H*), 8.05 (d, 1H, Ar*H*). ^13^C NMR (DMSO-*d*_6_, 293 K): δ 11.1 (*C*H_3_); 14.4 (*C*H_3_); 50.7 (N*C*H_2_Ph); 64.8, 66.3 (O*C*H_2_*C*H_2_O); 107.2 (4-*C*H); 111.3, 122.0, 123.3, 123.9, 128.0, 128.4, 130.0, 131.6, 133.6, 135.2, 141.7, 141.8, 144.2, 151.3 (ArH, Ar, *C*HCO, 3-*C*H and 5-*C*H); 161.8, 164.4 (*C*O). ^31^P{^1^H} NMR (DMSO-*d*_6_, 293 K): δ −144.20 (septet, *J*(F–P) = 710 Hz, PF_6_), −1.97 (sbr). ESI-MS (major positive ions, CH_3_CN), *m*/*z* (%): 587 (100) [Cu(PPh_3_)_2_]^+^, 659 (80) [Cu(L^2^)]^+^, 921 (10) [(PPh_3_)Cu(L^2^)]^+^. ESI-MS (major negative ions, CH_3_CN), *m*/*z* (%): 145 (100) [PF_6_]^−^. Calcd for C_65_H_58_Cl_2_CuF_6_N_6_O_4_P_3_: H, 4.40; C, 58.76; N, 6.33%. Found: H, 4.23; C, 58.42; N, 6.06%.

#### Synthesis of [(L^2^)CuCl_2_] (**13**)

CuCl_2_·2H_2_O (0.061 g, 0.359 mmol) was added to an acetonitrile solution (80 mL) of L^2^ (**2**, 0.214 g, 0.359 mmol). The reaction was stirred at room temperature for 24 h and dried under reduced pressure. Subsequently, the raw product was recrystallized using CH_3_CN/Et_2_O in a 1:10 ratio to obtain the dark green complex [(L^2^)CuCl_2_] (**13**) in 60% yield. mp: 148–150 °C. IR (cm^–1^): 3137mbr, 2960mbr, 2926mbr, 2856mbr (C–H); 1765m, 1716mbr (C=O); 1590m, 1563m (C=C/C=N); 1472m, 1442m, 1419m, 1386m, 1316m, 1273m, 1217s, 1162s, 1124s, 1105s, 1047s, 1007m, 954mbr, 899m, 858m, 835m, 788s, 752s, 721m, 708m. ESI-MS (major positive ions, CH_3_CN), *m*/*z* (%): 658 (100) [Cu(L^2^) – H]^+^. ESI-MS (major negative ions, CH_3_CN), *m*/*z* (%): 170 (100) [CuCl_3_]^−^. Calcd for C_29_H_28_Cl_4_CuN_6_O_4_: H, 3.87; C, 47.72; N, 11.51%. Found: H, 3.94; C, 47.48; N, 11.23%.

#### Synthesis of [(PTA)_2_Cu(L^4^)]PF_6_ (**14**)

This compound was prepared from L^4^ (**4**) following the procedure described for **5**. The reaction mixture was filtered, and the solution was dried under reduced pressure to obtain the white complex [(PTA)_2_Cu(L^4^)]PF_6_ (**14**) in 47% yield. mp: 227–230 °C. IR (cm^–1^): 3389wbr, 3252wbr (N–H); 2923wbr, 2887wbr (C–H); 1692m, 1658mbr (C=O); 1590w, 1562m, 1533mbr (C=C/C=N); 1493m, 1472m, 1449m, 1416m, 1388m, 1373m, 1314m, 1295mbr, 1267m, 1242m, 1230sh, 1176m, 1158sh, 1135w, 1104m, 1042mbr, 1013s, 968sbr, 948s, 894m, 874m, 833vs, 806sh, 776sh, 744s, 729s. ^1^H NMR (DMSO-*d*_6_, 293 K): δ 2.19 (s, 6H, C*H*_3_), 2.37 (s, 6H, C*H*_3_), 3.36–3.48 (m, 4H, NHC*H*_2_C*H*_2_NH), 4.04–4.66 (m, 24H, NC*H*_2_P and NC*H*_2_N), 5.82 (s, 2H, NC*H*_2_Ph), 6.10 (sbr, 2H, 4-C*H*), 6.76 (d, 1H, Ar*H*), 6.92 (s, 1H, C*H*CO), 7.33–7.79 (m, 5H, Ar*H*), 8.22 (d, 1H, Ar*H*), 8.43 (br, 1H, N*H*), 9.07 (br, 1H, N*H*). ^13^C NMR (DMSO-*d*_6_, 293 K): δ 11.1 (*C*H_3_); 14.4 (*C*H_3_); 38.3 (N*C*H_2_*C*H_2_N); 50.0 (N*C*H_2_Ph); 50.5, 51.9 (*C*H_2_P); 72.4 (*C*H_2_N); 107.0 (4-*C*H); 110.8, 122.5, 122.7, 123.2, 127.7, 128.3, 129.6, 130.6, 133.3, 133.7, 133.9, 138.5, 141.6, 142.3 (ArH, Ar, *C*HCO, 3-*C*H and 5-*C*H); 162.6, 164.2 (*C*O). ^31^P{^1^H}-NMR (DMSO-*d*_6_, 293 K): δ −144.02 (septet, *J*(F–P) = 714 Hz, PF_6_), −92.29 (s). ESI-MS (major positive ions, CH_3_CN), *m*/*z* (%): 657 (100) [Cu(L^4^)]^+^, 814 (25) [(PTA)Cu(L^4^)]^+^. ESI-MS (major negative ions, CH_3_CN), *m*/*z* (%): 145 (100) [PF_6_]^−^. Calcd for C_41_H_54_Cl_2_CuF_6_N_14_O_2_P_3_: H, 4.88; C, 44.11; N, 17.57%. Found: H, 4.61; C, 43.80; N, 17.21%.

#### Synthesis of [(PPh_3_)_2_Cu(L^4^)]PF_6_ (**15**)

This compound was prepared from PPh_3_ and L^4^ (**4**) following the procedure described for **5**. The reaction mixture was filtered, and the solution was dried under reduced pressure. Then, the residue in the round-bottom flask was solubilized with CHCl_3_ (10 mL) and filtered, and the solution was dried under reduced pressure to give the white complex [(PPh_3_)_2_Cu(L^4^)]PF_6_ (**15**) in 54% yield. mp: 100–105 °C. IR (cm^–1^): 3401wbr, 3284wbr (N–H); 3056wbr, 2926wbr, 2869wbr (C–H); 1694sh, 1672mbr (C=O); 1587m, 1562m, 1535m (C=C/C=N); 1492m, 1476m, 1435s, 1388m, 1311m, 1271m, 1230m, 1177m, 1158m, 1096m, 1071m, 1048m, 1028m, 998m, 918w, 835vs, 742vs. ^1^H NMR (DMSO-*d*_6_, 293 K): δ 1.82 (sbr, 6H, C*H*_3_), 2.43 (s, 6H, C*H*_3_), 3.37–3.41 (m, 4H, NHC*H*_2_C*H*_2_NH), 5.80 (s, 2H, NC*H*_2_Ph), 6.08 (s, 2H, 4-C*H*), 6.79 (d, 1H, Ar*H*), 7.07 (s, 1H, C*H*CO), 7.26–7.52 (m, 33H, Ar*H*), 7.73 (d, 1H, Ar*H*), 7.79 (d, 1H, Ar*H*), 8.20 (d, 1H, Ar*H*), 8.38 (t, 1H, N*H*), 9.35 (tbr, 1H, N*H*). ^13^C NMR (DMSO-*d*_6_, 293 K): δ 11.0 (*C*H_3_); 13.8 (*C*H_3_); 37.6 (N*C*H_2_*C*H_2_N); 50.0 (N*C*H_2_Ph); 107.2 (4-*C*H); 110.8, 122.5, 122.8, 123.2, 127.7, 128.2, 129.4, 129.6, 129.8, 130.8, 131.9, 132.0, 132.5, 133.4, 133.7, 133.8, 138.5, 141.6, 143.0, 150.5 (ArH, Ar, *C*HCO, 3-*C*H and 5-*C*H); 162.6, 164.5 (*C*O). ^31^P{^1^H} NMR (DMSO-*d*_6_, 293 K): δ −144.20 (septet, *J*(F–P) = 711 Hz, PF_6_), 4.99 (s). ^31^P{^1^H} NMR (CD_3_CN, 293 K): δ −143.53 (septet, *J*(F–P) = 705 Hz, PF_6_), −1.06 (sbr). ESI-MS (major positive ions, CH_3_CN), *m*/*z* (%): 587 (100) [Cu(PPh_3_)_2_]^+^, 657 (15) [Cu(L^4^)]^+^, 919 (45) [(PPh_3_)Cu(L^4^)]^+^. ESI-MS (major negative ions, CH_3_CN), *m*/*z* (%): 145 (100) [PF_6_]^−^. Calcd for C_65_H_60_Cl_2_CuF_6_N_8_O_2_P_3_: H, 4.56; C, 58.85; N, 8.45%. Found: H, 4.27; C, 58.51; N, 8.12%.

#### Synthesis of [(L^4^)CuCl_2_] (**16**)

This compound was prepared from L^4^ (**4**) following the procedure described for **7**, using CH_3_OH as a solvent, to give the light blue complex [(L^4^)CuCl_2_] (**16**) in 83% yield. mp: 248–250 °C. IR (cm^–1^): 3426wbr (N–H); 3169wbr, 2904mbr, 2799mbr (C–H); 1669s, 1620sh (C=O); 1576m, 1559s (C=C/C=N); 1528s, 1493m, 1467m, 1448m, 1428m, 1415m, 1397m, 1384m, 1375m, 1349m, 1314m, 1268w, 1248m, 1220m, 1194m, 1177m, 1157m, 1133m, 1116m, 1103m, 1064m, 1042m, 1005m, 987m, 949w, 913m, 875m, 861m, 837s, 807m, 787s, 754s, 744s, 708m. ESI-MS (major positive ions, CH_3_CN), *m*/*z* (%): 1248 (100) [Cu(L^4^)_2_ – H]^+^. ESI-MS (major negative ions, CH_3_CN), *m*/*z* (%): 170 (100) [CuCl_3_]^−^. Calcd for C_29_H_30_Cl_4_CuN_8_O_2_: H, 4.15; C, 47.85; N, 15.39%. Found: H, 4.43; C, 48.02; N, 15.09%.

### Spectroscopic Methods

#### Synchrotron Radiation-Induced X-ray Photoelectron Spectroscopy

SR-XPS measurements were performed at the Materials Science Beamline (MSB) at the ELETTRA synchrotron radiation source (Trieste, Italy). The MSB is placed at the left end of the bending magnet 6.1, and it is equipped with a plane grating monochromator that provides light in the energy range of 21–1000 eV. The base pressure in the UHV end-station is of 2 × 10^–10^ mbar; the end-station is equipped with a SPECS PHOIBOS 150 hemispherical electron analyzer, low-energy electron diffraction optics, a dual-anode Mg/Al X-ray source, an ion gun, and a sample manipulator with a K-type thermocouple attached to the rear side of the sample. For this experiment, we detected photoelectrons emitted by C 1s, N 1s, Cl 2p, Cu 2p, and O 1s core levels at the normal emission geometry. A photon energy of 650 eV impinging at 60° was selected for all signals, with the aim to maximize especially the N 1s signal intensity; Cu 2p spectra were also collected using the Al Kα anode source (1487.0 eV) so as to maximize its photoemission signal. Charging correction of BEs was always performed using the aromatic C 1s signal as a reference (BE 284.70 eV).^80^ To fit core-level spectra, we subtracted a Shirley background and then used Gaussian peak functions as signal components.^81,82^ The BE resolution was 0.6 eV for all measured core levels.

#### NEXAFS Spectroscopy

NEXAFS spectroscopy experiments were performed at the ELETTRA storage ring at the BEAR (Bending Magnet for Emission Absorption and Reflectivity) beamline, installed at the left exit of the 8.1 bending magnet exit. The apparatus is based on a bending magnet as a source, a beamline optics delivering photons from 5 eV up to about 1600 eV with a selectable degree of ellipticity. The carbon and nitrogen K-edge spectra were collected at grazing (20°), magic (54.7°), and normal (90°) incidence angles of the linearly polarized photon beam with respect to the sample surface. The photon energy and resolution were calibrated and experimentally tested at the K absorption edges of Ar, N_2_, and Ne. The normalization procedure consists of three steps: (i) the energy calibration, in which the I_0_ reference current (drain current) of the sample is shifted on the I_0_ reference current (drain current) of the Au clean sample recorded; (ii) the signal is obtained from the double ratio  after the interpolation of each signal to the Au reference. The double ratio allows us to correct for variations of the incident X-ray intensity with time as a function of photon energy due to instabilities of the electron beam in the storage ring or to changes of the X-ray optics in the beamline;^62^ and (iii) finally, the signal is reduced to the standard form through a pre-edge and post-edge fit: a linear pre-edge background is subtracted from the data and a linear post-edge fit is applied to the post-edge region to evaluate the jump and obtain the normalized signal.

#### X-ray Absorption Fine Structure

XAFS Cu K-edge (*E*_Cu_ = 8980 eV) experiments were performed at the XAFS beamline^83^ of the ELETTRA (Trieste) storage ring (complexes **10** and **16**) and the B18 beamline^84^ of the Diamond Light Source (UK) (complex **15**). Complexes **10** and **16** were measured in the transmission geometry at LN temperature. Samples were prepared in the form of solid homogeneous pellets, in which the weight ratio between the PVP matrix and the Cu complex sample was approximately 5:1. Complex **15** was measured in the fluorescence geometry using a 36 element Ge detector. The sample was homogeneously mixed with boron nitride, pressed into a pellet, and measured at 77 K using a liquid nitrogen cryostat. The data were treated using the standard procedure for data normalization and extraction of the structural EXAFS signal χ_exp_(*k*).^85^ The quantitative EXAFS data analysis was carried out using program FitEXA.^85^ The least squares method was used to minimize the difference between the experimental *k*^2^-weighted EXAFS function *k*^2^*χ*_exp_(*k*) and the theoretical one *k*^2^χ_teo_(*k*) in the reciprocal space 3 to 15 k^–1^. The model function was built as a sum of Gaussian-shaped shells, representative of the neighbor atoms around the absorber
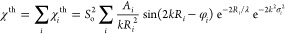


Each shell is defined by three parameters: the number of neighbors *N* (multiplicity), the average distance *R*_*i*_, and the MSRD parameter σ_*i*_^2^, representing the variance of the scattering (SS or MS) path distribution. The S_o_^2^ parameter, taking into account many body losses, was fixed to be 0.9. The same energy shift was used for all the contributions. The amplitude *A*_*i*_, phase shift φ_*i*_, and mean free path λ functions were calculated using FEFF 8.2 program^86^ using the molecular geometry around the absorber.

### Experiments with Cultured Human Cancer Cells

Cu(I) and Cu(II) complexes and the corresponding uncoordinated ligands and precursors were dissolved in DMSO just before the experiment, and a calculated amount of drug solution was added to the cell growth medium to a final solvent concentration of 0.5%, which had no detectable effects on cell viability. Cisplatin (Sigma Chemical Co, St. Louis, USA) and oxaliplatin (Sigma Chemical Co.) were dissolved in 0.9% sodium chloride solution.

### Cell Cultures

Human lung (H157), colon (HCT-15 and LoVo), and pancreatic (PSN-1) carcinoma cells were obtained from the American Type Culture Collection (ATCC, Rockville, MD, USA). Human cervical carcinoma A431 cells were kindly provided by Prof. F. Zunino (Division of Experimental Oncology B, Istituto Nazionale dei Tumori, Milan). Human ovarian adenocarcinoma 2008 cells were kindly provided by Prof. G. Marverti (Dipartimento di Scienze Biomediche, Università di Modena University, Italy). Human colon cancer cells resistant to doxorubicin (LoVo MDR) were kindly provided by Prof. M. P. Rigobello (Dipartimento di Scienze Biomediche, Università di Padova, Italy). The LoVo-OXP cells were derived, using a standard protocol, by growing LoVo cells in increasing concentrations of OXP and following 17 months of selection of resistant clones, as previously described.^23^

Cell lines were maintained in the logarithmic phase at 37 °C in a 5% carbon dioxide atmosphere using the following culture media containing 10% fetal calf serum (EuroClone, Milan, Italy), antibiotics (50 units/mL penicillin and 50 μg/mL streptomycin), and 2 mM l-glutamine: (i) RPMI-1640 medium (EuroClone) for A431, PSN-1, H157, HCT-15, and 2008 cells and (ii) Ham’S F-12 (Sigma Chemical Co.) for LoVo, LoVo MDR, and LoVo-OXP cells.

### MTT Assay

The growth inhibitory effect toward tumor cells was evaluated by means of an MTT assay.^23^ Briefly, 3–8 × 10^3^ cells/well, dependent upon the growth characteristics of the cell line, were seeded in 96-well microplates in growth medium (100 μL). After 24 h, the medium was removed and replaced with a fresh one containing the compound to be studied at the appropriate concentration. Triplicate cultures were established for each treatment. After 72 h, each well was treated with 10 μL of a 5 mg/mL MTT saline solution, and following 5 h of incubation, 100 μL of a sodium dodecyl sulfate solution in HCl 0.01 M was added. After overnight incubation, cell growth inhibition was detected by measuring the absorbance of each well at 570 nm using a Bio-Rad 680 microplate reader. Mean absorbance for each drug dose was expressed as a percentage of the control untreated well absorbance and plotted versus drug concentration. IC_50_ values, the drug concentrations that reduce the mean absorbance at 570 nm to 50% of those in the untreated control wells, were calculated using the four-parameter logistic (4-PL) model. Evaluation was based on the mean from at least four independent experiments.

### Spheroid Cultures

Spheroid cultures were obtained by seeding 2.5 × 10^3^ PSN-1 cancer cells/well in a round-bottom nontreated tissue culture 96-well plate (Greiner Bio-one, Kremsmünster, Austria) in phenol red free RPMI-1640 medium (Sigma Chemical Co.) containing 10% fetal calf serum and supplemented with 20% methyl cellulose stock solution.

### APH Assay

An APH modified assay was used for determining cell viability in 3D spheroids, as previously described.^87^ IC_50_ values were calculated using a 4-PL model.

### Copper Cellular Accumulation

PSN-1 cells (2.5 × 10^6^) were seeded in 75 cm^2^ flasks in growth medium (20 mL). After 24 h, the medium was replaced, and the cells were incubated for 24 h with tested complexes. Monolayers were then washed twice with ice-cold phosphate-buffered saline (PBS), harvested, and counted. Cell samples were subjected to five freeze–thaw cycles at −80 °C and then vigorously vortexed. The samples were treated with highly pure nitric acid and transferred into a microwave Teflon vessel. Samples were then submitted to standard mineralization procedures and analyzed for the copper amount by using a Varian AA Duo graphite furnace atomic absorption spectrometer (Varian, Palo Alto, CA; USA) at 324 nm. The calibration curve was obtained using known concentrations of standard solutions purchased from Sigma Chemical Co.

### ROS Production

The production of ROS was measured in PSN-1 cells (10^4^ per well) grown for 24 h in a 96-well plate in RPMI medium without phenol red (Sigma Chemical Co.). Cells were then washed with PBS and loaded with 10 μM 5-(and-6)-chloromethyl-2′,7′-dichlorodihydrofluorescein diacetate acetyl ester (CM-H_2_DCFDA) (Molecular Probes-Invitrogen, Eugene, OR) for 25 min in the dark. Afterward, cells were washed with PBS and incubated with increasing concentrations of tested compounds. Fluorescence increase was estimated utilizing a plate reader (Tecan Infinite M200 PRO, Männedorf, Switzerland) at 485 nm (excitation) and 527 nm (emission). Antimycin (3 μM, Sigma Chemical Co), a potent inhibitor of Complex III in the electron transport chain, and auranofin were used as positive controls.

### Quantification of Thiols

The PSN-1 cells (2 × 10^5^) were seeded in a six-well plate in growth medium (4 mL). After 24 h, cells were incubated for 24 h with increasing concentrations of tested compounds. Subsequently, the thiol content was measured as previously described.^88^

### Mitochondrial Membrane Potential

The mitochondrial membrane potential (ΔΨ) was assayed using the Mito-ID membrane potential kit according to the manufacturer’s instructions (Enzo Life Sciences, Farmingdale, NY). Briefly, PSN-1 cells (8 × 10^3^ per well) were seeded in 96-well plates; after 24 h, cells were washed with PBS and loaded with the Mito-ID detection reagent for 30 min at 37 °C in the dark. Afterward, cells were incubated with increasing concentrations of tested complexes. Fluorescence intensity was estimated using a plate reader (Tecan Infinite M200 PRO, Männedorf, Switzerland) at 490 (excitation) and 590 nm (emission). CCCP (4 μM, Sigma Chemical Co), a chemical inhibitor of the oxidative phosphorylation, was used as the positive control.

### Apoptosis Induction

PSN-1 cells were seeded into 8-well tissue culture slides (BD Falcon, Bedford, MA, USA) at 5 × 10^4^ cells/well (0.8 cm^2^). After 24 h, the cells were washed twice with PBS, and following 48 h of treatment with IC_50_ doses of tested compounds, cells were stained for 5 min with 10 μg/mL Hoechst 33258 [20-(4-hydroxyphenyl)-5-(4-methyl-1-piperazinyl)-2,50-bi-1*H*-benzimidazole trihydrochloride hydrate, Sigma-Aldrich, St. Louis, MI, USA] in PBS. Samples were examined at 5× and 40× magnification in a Zeiss LSM 800 confocal microscope using the Zeiss ZEN 2.3 software system.

### Statistical Analysis

All values are the means ± S.D. of no less than three measurements starting from three different cell cultures. Multiple comparisons were made using ANOVA, followed by the Tukey–Kramer multiple comparison test (*P < 0.05, **P < 0.01), using GraphPad software.

## Supporting Information Available

EXAFS data analysis details; XPS data analysis results: BE, FWHM, atomic percents, and proposed assignments; XPS Cu 2p spectra of Cu(II) complex **10** (A) and Cu(I) complex **15** (B); and stability studies, where all complexes were dissolved at 50 μM in 0.5% DMSO/RPMI medium and UV–visible spectra were recorded at *t* = 0 min and *t* = 1440 min = 24 h (PDF)

## References

[ref1] Nath K.; Guo L.; Nancolas B.; Nelson D. S.; Shestov A. A.; Lee S.-C.; Roman J.; Zhou R.; Leeper D. B.; Halestrap A. P.; Blair I. A.; Glickson J. D. Mechanism of antineoplastic activity of lonidamine. Biochim. Biophys. Acta, Rev. Cancer 2016, 1866, 151–162. 10.1016/j.bbcan.2016.08.001. PMC5138080 27497601

[ref2] Cervantes-Madrid D.; Romero Y.; Dueñas-González A. Reviving Lonidamine and 6-Diazo-5-oxo-L-norleucine to Be Used in Combination for Metabolic Cancer Therapy. BioMed Res. Int. 2015, 2015, 690492 10.1155/2015/690492. 26425550 PMC4575731

[ref3] Guo L.; Shestov A. A.; Worth A. J.; Nath K.; Nelson D. S.; Leeper D. B.; Glickson J. D.; Blair I. A. Inhibition of Mitochondrial Complex II by the Anticancer Agent Lonidamine. J. Biol. Chem. 2016, 291, 42–57. 10.1074/jbc.M115.697516. 26521302 PMC4697178

[ref4] Sadeghi R. N.; Karami-Tehrani F.; Salami S. Targeting prostate cancer cell metabolism: impact of hexokinase and CPT-1 enzymes. Tumor Biol. 2015, 36, 2893–2905. 10.1007/s13277-014-2919-4. 25501281

[ref5] Nancolas B.; Guo L.; Zhou R.; Nath K.; Nelson D. S.; Leeper D. B.; Blair I. A.; Glickson J. D.; Halestrap A. P. The anti-tumour agent lonidamine is a potent inhibitor of the mitochondrial pyruvate carrier and plasma membrane monocarboxylate transporters. Biochem. J. 2016, 473, 929–936. 10.1042/bj20151120. 26831515 PMC4814305

[ref6] Nath K.; Nelson D. S.; Heitjan D. F.; Leeper D. B.; Zhou R.; Glickson J. D. Lonidamine induces intracellular tumor acidification and ATP depletion in breast, prostate and ovarian cancer xenografts and potentiates response to doxorubicin. NMR Biomed. 2015, 28, 281–290. 10.1002/nbm.3240. 25504852 PMC4361034

[ref7] Ravagnan L.; Marzo I.; Costantini P.; Susin S. A.; Zamzami N.; Petit P. X.; Hirsch F.; Goulbern M.; Poupon M.-F.; Miccoli L.; Xie Z.; Reed J. C.; Kroemer G. Lonidamine triggers apoptosis via a direct, Bcl-2-inhibited effect on the mitochondrial permeability transition pore. Oncogene 1999, 18, 2537–2546. 10.1038/sj.onc.1202625. 10353597

[ref8] Crompton M. The mitochondrial permeability transition pore and its role in cell death. Biochem. J. 1999, 341, 233–249. 10.1042/bj3410233. 10393078 PMC1220352

[ref9] Rosbe K. W.; Brann T. W.; Holden S. A.; Teicher B. A.; Frei E. Effect of Lonidamine on the cytotoxicity of four alkylating agents in vitro. Cancer Chemother. Pharmacol. 1989, 25, 32–36. 10.1007/bf00694335. 2590999

[ref10] Angioli R.; Janicek M.; Sevin B.; Estape R.; Averette H.; Koechli O.; Untch M.; Penalver M. Use of lonidamine to potentiate the effect of cisplatin and carboplatin on platinum resistant human ovarian cancer cells. Internet J. Oncol. 1997, 11, 777–780. 10.3892/ijo.11.4.777. 21528274

[ref11] Chen H.; Chen F.; Hu W.; Gou S. Effective platinum(IV) prodrugs conjugated with lonidamine as a functional group working on the mitochondria. J. Inorg. Biochem. 2018, 180, 119–128. 10.1016/j.jinorgbio.2017.11.017. 29253663

[ref12] Okulova Y. N.; Zenin I. V.; Shutkov I. A.; Kirsanov K. I.; Kovaleva O. N.; Lesovaya E. A.; Fetisov T. I.; Milaeva E. R.; Nazarov A. A. Antiproliferative activity of Pt(IV) complexes with lonidamine and bexarotene ligands attached via succinate-ethylenediamine linker. Inorg. Chim. Acta 2019, 495, 119010 10.1016/j.ica.2019.119010.

[ref13] Nosova Y. N.; Foteeva L. S.; Zenin I. V.; Fetisov T. I.; Kirsanov K. I.; Yakubovskaya M. G.; Antonenko T. A.; Tafeenko V. A.; Aslanov L. A.; Lobas A. A.; Gorshkov M. V.; Galanski M.; Keppler B. K.; Timerbaev A. R.; Milaeva E. R.; Nazarov A. A. Enhancing the Cytotoxic Activity of Anticancer Pt IV Complexes by Introduction of Lonidamine as an Axial Ligand. Eur. J. Med. Chem. 2017, 2017, 1785–1791. 10.1002/ejic.201600857.

[ref14] Ruttala H. B.; Ramasamy T.; Poudel B. K.; Ruttala R. R. T.; Jin S. G.; Choi H.-G.; Ku S.-K.; Yong C. S.; Kim J. O. Multi-responsive albumin-lonidamine conjugated hybridized gold nanoparticle as a combined photothermal-chemotherapy for synergistic tumor ablation. Acta Biomater. 2020, 101, 531–543. 10.1016/j.actbio.2019.11.003. 31706039

[ref15] Qin Q.-P.; Liu Y.-C.; Wang H.-L.; Qin J.-L.; Cheng F.-J.; Tang S.-F.; Liang H. Synthesis and antitumor mechanisms of a copper(ii) complex of anthracene-9-imidazoline hydrazone (9-AIH). Metallomics 2015, 7, 1124–1136. 10.1039/C5MT00027K. 25904543

[ref16] Raman N.; Jeyamurugan R.; Senthilkumar R.; Rajkapoor B.; Franzblau S. G. In vivo and in vitro evaluation of highly specific thiolate carrier group copper(II) and zinc(II) complexes on Ehrlich ascites carcinoma tumor model. Eur. J. Med. Chem. 2010, 45, 5438–5451. 10.1016/j.ejmech.2010.09.004. 20864225

[ref17] Palanimuthu D.; Shinde S. V.; Somasundaram K.; Samuelson A. G. In Vitro and in Vivo Anticancer Activity of Copper Bis(thiosemicarbazone) Complexes. J. Med. Chem. 2013, 56, 722–734. 10.1021/jm300938r. 23320568

[ref18] Montagner D.; Fresch B.; Browne K.; Gandin V.; Erxleben A. A Cu(ii) complex targeting the translocator protein: in vitro and in vivo antitumor potential and mechanistic insights. Chem. Commun. 2017, 53, 134–137. 10.1039/C6CC08100B. 27924322

[ref19] Laws K.; Bineva-Todd G.; Eskandari A.; Lu C.; O’Reilly N.; Suntharalingam K. A Copper(II) Phenanthroline Metallopeptide That Targets and Disrupts Mitochondrial Function in Breast Cancer Stem Cells. Angew. Chem., Int. Ed. 2018, 57, 287–291. 10.1002/ange.201710910. 29144008

[ref20] Mahendiran D.; Kumar R. S.; Viswanathan V.; Velmurugan D.; Rahiman A. K. In vitro and in vivo anti-proliferative evaluation of bis(4′-(4-tolyl)-2,2′:6′,2″-terpyridine)copper(II) complex against Ehrlich ascites carcinoma tumors. JBIC, J. Biol. Inorg. Chem. 2017, 22, 1109–1122. 10.1007/s00775-017-1488-6. 28884428

[ref21] Becco L.; García-Ramos J. C.; Azuara L. R.; Gambino D.; Garat B. Analysis of the DNA Interaction of Copper Compounds Belonging to the Casiopeínas Antitumoral Series. Biol. Trace Elem. Res. 2014, 161, 210–215. 10.1007/s12011-014-0098-1. 25119709

[ref22] Gandin V.; Ceresa C.; Esposito G.; Indraccolo S.; Porchia M.; Tisato F.; Santini C.; Pellei M.; Marzano C. Therapeutic potential of the phosphino Cu(I) complex (HydroCuP) in the treatment of solid tumors. Sci. Rep. 2017, 7, 13936 10.1038/s41598-017-13698-1. 29066771 PMC5655689

[ref23] Gandin V.; Pellei M.; Tisato F.; Porchia M.; Santini C.; Marzano C. A novel copper complex induces paraptosis in colon cancer cells via the activation of ER stress signalling. J. Cell. Mol. Med. 2012, 16, 142–151. 10.1111/j.1582-4934.2011.01292.x. 21388518 PMC3823100

[ref24] Gandin V.; Tisato F.; Dolmella A.; Pellei M.; Santini C.; Giorgetti M.; Marzano C.; Porchia M. In Vitro and in Vivo Anticancer Activity of Copper(I) Complexes with Homoscorpionate Tridentate Tris(pyrazolyl)borate and Auxiliary Monodentate Phosphine Ligands. J. Med. Chem. 2014, 57, 4745–4760. 10.1021/jm500279x. 24793739

[ref25] Erxleben A. Interactions of copper complexes with nucleic acids. Coord. Chem. Rev. 2018, 360, 92–121. 10.1016/j.ccr.2018.01.008.

[ref26] Krasnovskaya O.; Naumov A.; Guk D.; Gorelkin P.; Erofeev A.; Beloglazkina E.; Majouga A. Copper Coordination Compounds as Biologically Active Agents. Int. J. Mol. Sci. 2020, 21, 3965 10.3390/ijms21113965. PMC7312030 32486510

[ref27] Kellett A.; Molphy Z.; McKee V.; Slator C. In Metal-based Anticancer Agents; Casini A., Vessières A., Meier-Menches S. M., Eds.; The Royal Society of Chemistry, 2019; pp 91–119.

[ref28] Shao S.; Si J.; Shen Y. Copper as the Target for Anticancer Nanomedicine. Adv. Ther. 2019, 2, 1800147 10.1002/adtp.201800147.

[ref29] Balsano C.; Porcu C.; Sideri S. Is copper a new target to counteract the progression of chronic diseases?. Metallomics 2018, 10, 1712–1722. 10.1039/c8mt00219c. 30339169

[ref30] Denoyer D.; Masaldan S.; La Fontaine S.; Cater M. A. Targeting copper in cancer therapy: “Copper That Cancer”. Metallomics 2015, 7, 1459–1476. 10.1039/C5MT00149H. 26313539

[ref31] Gandin V.; Trenti A.; Porchia M.; Tisato F.; Giorgetti M.; Zanusso I.; Trevisi L.; Marzano C. Homoleptic phosphino copper(i) complexes with in vitro and in vivo dual cytotoxic and anti-angiogenic activity. Metallomics 2015, 7, 1497–1507. 10.1039/C5MT00163C. 26190698

[ref32] Silva-Platas C.; Guerrero-Beltrán C. E.; Carrancá M.; Castillo E. C.; Bernal-Ramírez J.; Oropeza-Almazán Y.; González L. N.; Rojo R.; Martínez L. E.; Valiente-Banuet J.; Ruiz-Azuara L.; Bravo-Gómez M. E.; García N.; Carvajal K.; García-Rivas G. Antineoplastic copper coordinated complexes (Casiopeinas) uncouple oxidative phosphorylation and induce mitochondrial permeability transition in cardiac mitochondria and cardiomyocytes. J. Bioenerg. Biomembr. 2016, 48, 43–54. 10.1007/s10863-015-9640-x. 26739598

[ref33] Weekley C. M.; He C. Developing drugs targeting transition metal homeostasis. Curr. Opin. Chem. Biol. 2017, 37, 26–32. 10.1016/j.cbpa.2016.12.011. 28040658 PMC5947866

[ref34] Wehbe M.; Leung A. W. Y.; Abrams M. J.; Orvig C.; Bally M. B. A Perspective—can copper complexes be developed as a novel class of therapeutics?. Dalton Trans. 2017, 46, 10758–10773. 10.1039/C7DT01955F. 28702645

[ref35] Baldari S.; Di Rocco G.; Toietta G. Current Biomedical Use of Copper Chelation Therapy. Int. J. Mol. Sci. 2020, 21, 1069 10.3390/ijms21031069. PMC7037088 32041110

[ref36] Santini C.; Pellei M.; Gandin V.; Porchia M.; Tisato F.; Marzano C. Advances in Copper Complexes as Anticancer Agents. Chem. Rev. 2014, 114, 815–862. 10.1021/Cr400135x. 24102434

[ref37] Lelièvre P.; Sancey L.; Coll J.-L.; Deniaud A.; Busser B. The Multifaceted Roles of Copper in Cancer: A Trace Metal Element with Dysregulated Metabolism, but Also a Target or a Bullet for Therapy. Cancers 2020, 12, 3594 10.3390/cancers12123594. PMC7760327 33271772

[ref38] Medici S.; Peana M.; Nurchi V. M.; Lachowicz J. I.; Crisponi G.; Zoroddu M. A. Noble metals in medicine: Latest advances. Coord. Chem. Rev. 2015, 284, 329–350. 10.1016/j.ccr.2014.08.002.

[ref39] Tisato F.; Marzano C.; Porchia M.; Pellei M.; Santini C. Copper in Diseases and Treatments, and Copper-Based Anticancer Strategies. Med. Res. Rev. 2010, 30, 708–749. 10.1002/Med.20174. 19626597

[ref40] Marzano C.; Pellei M.; Tisato F.; Santini C. Copper complexes as anticancer agents. Anti-Cancer Agents Med. Chem. 2009, 9, 185–211. 10.2174/187152009787313837. 19199864

[ref41] Zehra S.; Tabassum S.; Arjmand F. Biochemical pathways of copper complexes: progress over the past 5 years. Drug Discovery Today 2021, 26, 1086–1096. 10.1016/j.drudis.2021.01.015. 33486113

[ref42] Singh N. K.; Kumbhar A. A.; Pokharel Y. R.; Yadav P. N. Anticancer potency of copper(II) complexes of thiosemicarbazones. J. Inorg. Biochem. 2020, 210, 111134 10.1016/j.jinorgbio.2020.111134. 32673842

[ref43] Allardyce C. S.; Dyson P. J. Metal-based drugs that break the rules. Dalton Trans. 2016, 45, 3201–3209. 10.1039/C5DT03919C. 26820398

[ref44] Spreckelmeyer S.; Orvig C.; Casini A. Cellular Transport Mechanisms of Cytotoxic Metallodrugs: An Overview beyond Cisplatin. Molecules 2014, 19, 15584–15610. 10.3390/molecules191015584. 25268716 PMC6271550

[ref45] Zaki M.; Arjmand F.; Tabassum S. Current and future potential of metallo drugs: Revisiting DNA-binding of metal containing molecules and their diverse mechanism of action. Inorg. Chim. Acta 2016, 444, 1–22. 10.1016/j.ica.2016.01.006.

[ref46] Molinaro C.; Martoriati A.; Pelinski L.; Cailliau K. Copper Complexes as Anticancer Agents Targeting Topoisomerases I and II. Cancers 2020, 12, 2863 10.3390/cancers12102863. PMC7601307 33027952

[ref47] Zanella A.; Gandin V.; Porchia M.; Refosco F.; Tisato F.; Sorrentino F.; Scutari G.; Rigobello M. P.; Marzano C. Cytotoxicity in human cancer cells and mitochondrial dysfunction induced by a series of new copper(I) complexes containing tris(2-cyanoethyl)phosphines. Invest. New Drugs 2011, 29, 1213–1223. 10.1007/s10637-010-9466-7. 20567997

[ref48] Otero A.; Fernández-Baeza J.; Lara-Sánchez A.; Sánchez-Barba L. F. Metal complexes with heteroscorpionate ligands based on the bis(pyrazol-1-yl)methane moiety: Catalytic chemistry. Coord. Chem. Rev. 2013, 257, 1806–1868. 10.1016/j.ccr.2013.01.027.

[ref49] Pellei M.; Papini G.; Trasatti A.; Giorgetti M.; Tonelli D.; Minicucci M.; Marzano C.; Gandin V.; Aquilanti G.; Dolmella A.; Santini C. Nitroimidazole and glucosamine conjugated heteroscorpionate ligands and related copper(II) complexes. Syntheses, biological activity and XAS studies. Dalton Trans. 2011, 40, 9877–9888. 10.1039/c1dt10486a. 21709917

[ref50] Giorgetti M.; Tonelli S.; Zanelli A.; Aquilanti G.; Pellei M.; Santini C. Synchrotron radiation X-ray absorption spectroscopic studies in solution and electrochemistry of a nitroimidazole conjugated heteroscorpionate copper(II) complex. Polyhedron 2012, 48, 174–180. 10.1016/j.poly.2012.08.073.

[ref51] Morelli M. B.; Amantini C.; Santoni G.; Pellei M.; Santini C.; Cimarelli C.; Marcantoni E.; Petrini M.; Del Bello F.; Giorgioni G.; Piergentili A.; Quaglia W. Novel antitumor copper(ii) complexes designed to act through synergistic mechanisms of action, due to the presence of an NMDA receptor ligand and copper in the same chemical entity. New J. Chem. 2018, 42, 11878–11887. 10.1039/c8nj01763h.

[ref52] Pellei M.; Bagnarelli L.; Luciani L.; Del Bello F.; Giorgioni G.; Piergentili A.; Quaglia W.; De Franco M.; Gandin V.; Marzano C.; Santini C. Synthesis and Cytotoxic Activity Evaluation of New Cu(I) Complexes of Bis(pyrazol-1-yl) Acetate Ligands Functionalized with an NMDA Receptor Antagonist. Int. J. Mol. Sci. 2020, 21, 2616 10.3390/ijms21072616. PMC7178194 32283777

[ref53] Schiesaro I.; Venditti I.; Pellei M.; Santini C.; Bagnarelli L.; Iucci G.; Battocchio C.; Meneghini C. Metal Coordination Core in Copper(II) Complexes Investigated by XAFS. Synchrotron Radiation Science and Applications; Springer Proceedings in Physics; Springer, 2021; Vol. 2021; pp 169–179.

[ref54] Beck A.; Weibert B.; Burzlaff N. MonoanionicN,N,O-Scorpionate Ligands and their Iron(II) and Zinc(II) Complexes: Models for Mononuclear Active Sites of Non-Heme Iron Oxidases and Zinc Enzymes. Eur. J. Inorg. Chem. 2001, 2001, 521–527. 10.1002/1099-0682(200102)2001:2<521::aid-ejic521>3.0.co;2-q.

[ref55] Burzlaff N.; Hegelmann I.; Weibert B. Bis(pyrazol-1-yl)acetates as tripodal “scorpionate” ligands in transition metal carbonyl chemistry: syntheses, structures and reactivity of manganese and rhenium carbonyl complexes of the type [LM(CO)3] (L = bpza, bdmpza). J. Organomet. Chem. 2001, 626, 16–23. 10.1016/s0022-328x(01)00648-9.

[ref56] Clark D. T.; Lilley D. M. J. Molecular core binding energies for some five membered ring heterocycles as determined by X-ray photoelectron spectroscopy. Chem. Phys. Lett. 1971, 9, 234–237. 10.1016/0009-2614(71)85038-8.

[ref57] Polzonetti G.; Battocchio C.; Goldoni A.; Larciprete R.; Carravetta V.; Paolesse R.; Russo M. V. Interface formation between C60 and diethynyl-Zn-porphyrinato investigated by SR-induced photoelectron and near-edge X-ray absorption (NEXAFS) spectroscopies. Chem. Phys. 2004, 297, 307–314. 10.1016/j.chemphys.2003.10.024.

[ref58] Battocchio C.; Fratoddi I.; Iucci G.; Russo M. V.; Goldoni A.; Parent P.; Polzonetti G. Dinuclear Pt and Pd complexes with metalloporphyrin bridges: A NEXAFS study of the electronic structure and self-assembling properties. Mater. Sci. Eng., C 2007, 27, 1338–1342. 10.1016/j.msec.2006.06.014.

[ref59] Gabrielli S.; Pellei M.; Venditti I.; Fratoddi I.; Battocchio C.; Iucci G.; Schiesaro I.; Meneghini C.; Palmieri A.; Marcantoni E.; Bagnarelli L.; Vallesi R.; Santini C. Development of new and efficient copper(II) complexes of hexyl bis(pyrazolyl)acetate ligands as catalysts for allylic oxidation. Dalton Trans. 2020, 49, 15622–15632. 10.1039/d0dt02952a. 33095220

[ref60] NIST X-ray Photoelectron Spectroscopy Database, version 4.1; National Institute of Standards and Technology. http://srdata.nist.gov/xps/.=NIST.

[ref61] Klein J. C.; Li C. P.; Hercules D. M.; Black J. F. Decomposition of Copper Compounds in X-Ray Photoelectron Spectrometers. Appl. Spectrosc. 1984, 38, 729–734. 10.1366/0003702844555016.

[ref62] Stöhr J. NEXAFS Spectroscopy; Springer-Verlag Berlin HeidelbergBerlin Heidelberg GmbH, 1992; Vol. 25.

[ref63] Syugaev A. V.; Maratkanova A. N.; Smirnov D. A. Molecular orientation in electrodeposited polypyrrole films. J. Solid State Electrochem. 2018, 22, 2127–2134. 10.1007/s10008-018-3925-z.

[ref64] Pavlychev A. A.; Hallmeier K. H.; Hennig C.; Hennig L.; Szargan R. Nitrogen K-shell excitations in complex molecules and polypyrrole. Chem. Phys. 1995, 201, 547–555. 10.1016/0301-0104(95)00287-1.

[ref65] Franchi S.; Secchi V.; Santi M.; Dettin M.; Zamuner A.; Battocchio C.; Iucci G. Biofunctionalization of TiO2 surfaces with self-assembling oligopeptides in different pH and Ionic Strength conditions: Charge effects and molecular organization. Mater. Sci. Eng., C 2018, 90, 651–656. 10.1016/j.msec.2018.05.006. 29853135

[ref66] Benfatto M.; Meneghini C. In Synchrotron Radiation: Basics, Methods and Applications; Mobilio S., Boscherini F., Meneghini C., Eds.; Springer Berlin Heidelberg: Berlin, Heidelberg, 2015; pp 213–240.

[ref67] Chaboy J.; Muñoz-Páez A.; Carrera F.; Merkling P.; Marcos E. S. Ab initiox-ray absorption study of copperK-edge XANES spectra in Cu(II) compounds. Phys. Rev. B: Condens. Matter Mater. Phys. 2005, 71, 134208 10.1103/PhysRevB.71.134208.

[ref68] Giorgetti M.; Guadagnini L.; Fiddy S. G.; Santini C.; Pellei M. Cu K-edge EXAFS on copper(I) complexes containing dihydridobis(3-nitro-1,2,4-triazol-1-yl)borate and bis(1,2,4-triazol-1-yl)acetate ligand: Evidence for the Cu-O interaction. Polyhedron 2009, 28, 3600–3606. 10.1016/j.poly.2009.07.032.

[ref69] Kau L. S.; Spira-Solomon D. J.; Penner-Hahn J. E.; Hodgson K. O.; Solomon E. I. X-ray absorption edge determination of the oxidation state and coordination number of copper. Application to the type 3 site in Rhus vernicifera laccase and its reaction with oxygen. J. Am. Chem. Soc. 1987, 109, 6433–6442. 10.1021/ja00255a032.

[ref70] Fornasini P. Synchrotron Radiation: Basics, Methods and Applications; Springer Berlin Heidelberg, 2015; pp 181–211.

[ref71] Noordhuis P.; Laan A. C.; van de Born K.; Losekoot N.; Kathmann I.; Peters G. J. Oxaliplatin activity in selected and unselected human ovarian and colorectal cancer cell lines. Biochem. Pharmacol. 2008, 76, 53–61. 10.1016/j.bcp.2008.04.007. 18508032

[ref72] Wersinger C.; Rebel G.; Lelong-Rebel I. Detailed study of the different taurine uptake systems of colon LoVo MDR and non-MDR cell lines. Amino Acids 2000, 19, 667–685. 10.1007/s007260070015. 11140368

[ref73] Marzano C.; Pellei M.; Colavito D.; Alidori S.; Lobbia G. G.; Gandin V.; Tisato F.; Santini C. Synthesis, characterization, and in vitro antitumor properties of tris(hydroxymethyl)phosphine copper(I) complexes containing the new bis(1,2,4-triazol-1-yl)acetate ligand. J. Med. Chem. 2006, 49, 7317–7324. 10.1021/jm0601248. 17149861

[ref74] Pellei M.; Gandin V.; Cimarelli C.; Quaglia W.; Mosca N.; Bagnarelli L.; Marzano C.; Santini C. Syntheses and biological studies of nitroimidazole conjugated heteroscorpionate ligands and related Cu(I) and Cu(II) complexes. J. Inorg. Biochem. 2018, 187, 33–40. 10.1016/j.jinorgbio.2018.07.008. 30053534

[ref75] Zanoni M.; Cortesi M.; Zamagni A.; Arienti C.; Pignatta S.; Tesei A. Modeling neoplastic disease with spheroids and organoids. J. Hematol. Oncol. 2020, 13, 97 10.1186/s13045-020-00931-0. 32677979 PMC7364537

[ref76] Öhrvik H.; Aaseth J.; Horn N. Orchestration of dynamic copper navigation—new and missing pieces. Metallomics 2017, 9, 1204–1229. 10.1039/c7mt00010c. 28685789

[ref77] Xu H. N.; Feng M.; Nath K.; Nelson D.; Roman J.; Zhao H.; Lin Z.; Glickson J.; Li L. Z. Optical Redox Imaging of Lonidamine Treatment Response of Melanoma Cells and Xenografts. Mol. Imaging Biol. 2019, 21, 426–435. 10.1007/s11307-018-1258-z. 30151646 PMC6525077

[ref78] Nordberg J.; Arnér E. S. J. Reactive oxygen species, antioxidants, and the mammalian thioredoxin system1 1This review is based on the licentiate thesis “Thioredoxin reductase-interactions with the redox active compounds 1-chloro-2,4-dinitrobenzene and lipoic acid” by Jonas Nordberg, 2001, Karolinska Institute, Stockholm, ISBN 91-631-1064-4. Free Radical Biol. Med. 2001, 31, 1287–1312. 10.1016/s0891-5849(01)00724-9. 11728801

[ref79] Fontana F.; Raimondi M.; Marzagalli M.; Di Domizio A.; Limonta P. The emerging role of paraptosis in tumor cell biology: Perspectives for cancer prevention and therapy with natural compounds. Biochim. Biophys. Acta, Rev. Cancer 2020, 1873, 188338 10.1016/j.bbcan.2020.188338. 31904399

[ref80] Moulder J. F.; Stickle W. F.; Sobol P. E.; Bomben K. D. Handbook of X-Ray Photoelectron Spectroscopy; Eden Prairie, 1996.

[ref81] Beamson G.; Briggs D. In Surface and Interface Analysis; Watts J. F., Ed.; John Wiley & Sons: Chichester, 1992; Vol. 20, p 267.

[ref82] Shirley D. A. High-resolution x-ray photoemission spectrum of the valence bands of gold. Phys. Rev. B: Solid State 1972, 5, 4709–4714. 10.1103/PhysRevB.5.4709.

[ref83] Dent A. J.; Cibin G.; Ramos S.; Smith A. D.; Scott S. M.; Varandas L.; Pearson M. R.; Krumpa N. A.; Jones C. P.; Robbins P. E. B18: A core XAS spectroscopy beamline for Diamond. J. Phys.: Conf. Ser. 2009, 190, 012039 10.1088/1742-6596/190/1/012039.

[ref84] Cicco A. D.; Aquilanti G.; Minicucci M.; Principi E.; Novello N.; Cognigni A.; Olivi L. Novel XAFS capabilities at ELETTRA synchrotron light source. J. Phys.: Conf. Ser. 2009, 190, 012043 10.1088/1742-6596/190/1/012043.

[ref85] Meneghini C.; Bardelli F.; Mobilio S. ESTRA-FitEXA: A software package for EXAFS data analysis. Nucl. Instrum. Methods Phys. Res., Sect. B 2012, 285, 153–157. 10.1016/j.nimb.2012.05.027.

[ref86] Ankudinov A. L.; Ravel B.; Rehr J. J.; Conradson S. D. Real-space multiple-scattering calculation and interpretation of x-ray-absorption near-edge structure. Phys. Rev. B: Condens. Matter Mater. Phys. 1998, 58, 7565–7576. 10.1103/PhysRevB.58.7565.

[ref87] Carcelli M.; Tegoni M.; Bartoli J.; Marzano C.; Pelosi G.; Salvalaio M.; Rogolino D.; Gandin V. In vitro and in vivo anticancer activity of tridentate thiosemicarbazone copper complexes: Unravelling an unexplored pharmacological target. Eur. J. Med. Chem. 2020, 194, 112266 10.1016/j.ejmech.2020.112266. 32248006

[ref88] Rigobello M. P.; Folda A.; Citta A.; Scutari G.; Gandin V.; Fernandes A. P.; Rundlöf A.-K.; Marzano C.; Björnstedt M.; Bindoli A. Interaction of selenite and tellurite with thiol-dependent redox enzymes: Kinetics and mitochondrial implications. Free Radical Biol. Med. 2011, 50, 1620–1629. 10.1016/j.freeradbiomed.2011.03.006. 21397686

