# Supplementary material for: Cu(I) and Cu(II) Complexes Based on Lonidamine-Conjugated Ligands Designed to Promote Synergistic Antitumor Effects
Source: Inorg Chem. 2022 Mar 14;61(12):4919–37. doi: 10.1021/acs.inorgchem.1c03658 (PMC8965879; doi:10.1021/acs.inorgchem.1c03658)
Supplement: Supplementary file 1 — ic1c03658_si_001.pdf [file ic1c03658_si_001.pdf]

Supporting Information

Cu(I) and Cu(II) complexes based on lonidamine-conjugated ligands designed to promote synergistic antitumor effects

Fabio Del Bello,[a] Maura Pellei,*[b] Luca Bagnarelli,[b] Carlo Santini,[b] Gianfabio Giorgioni,[a] Alessandro Piergentili,[a] Wilma Quaglia,*[a] Chiara Battocchio,[c] Giovanna Iucci,[c] Irene Schiesaro,[c] Carlo Meneghini,[c] Iole Venditti,[c] Nitya Ramanan,[d] Michele De Franco,[e] Paolo Sgarbossa,[f] Cristina Marzano,[e] and Valentina Gandin[e]

[a]*School of Pharmacy, Medicinal Chemistry Unit, University of Camerino, via S. Agostino 1, 62032 Camerino, Italy*

[b]*School of Science and Technology, Chemistry Division, University of Camerino, via S. Agostino 1, 62032 Camerino, Italy*

[c]*Department of Science, Roma Tre University, Via della Vasca Navale 79, 00146, Roma, Italy*

[d]*Diamond Light Source, Harwell Science and Innovation Campus, Didcot, OX11 0DE, UK*

[e]*Department of Pharmaceutical and Pharmacological Sciences, University of Padova, via Marzolo 5, 35131 Padova, Italy*

[f]*Department of Industrial Engineering, University of Padova, via Marzolo 9, 35131 Padova, Italy*

EXAFS data analysis details

The experimental Cu K edge X-ray absorption spectra were treated along the standard procedures for data normalization and extraction of the structural EXAFS signal.[1] The quantitative EXAFS analysis was carried out using the atomic coordinates of the two residues as starting point for the data refinement that was carried out fitting the k^2 weighted structural signal $k^2\chi(k)$ in the k-space (without Fourier Filtering) in the 3-15 $Å^{-1}$ k-range. The appropriate single (SS) and multiple (MS) scattering paths were selected on the basis of their amplitude and statistical significance,[1] grouping in a single contribution those paths with similar geometry and composition. To reduce the correlation among the parameters the path multiplicity numbers were constrained to the molecular structure (see Table 2 of the manuscript). The experimental data, best fit and examples of the used paths are presented in Figure 4 of the manuscript. The differences among the spectra of the three samples (Figure 4A) can be understood in a more intuitive way looking at the FT moduli |FT| (Figure 4B): the complex **10** data present a major main peak around 1.5 Å (uncorrected for the phase shift), originating from 2 N at around 2 Å and 2 Cl at around 2.2 Å. In the next neighbour region the two main |FT| features at around 2.5 Å and 3.5 Å (uncorrected for the phase shift) are ascribed, one to N(2) and C neighbours from the pyrazole rings (around 3 Å), the other to the SS and MS paths to the C(2) atoms. These MS contributions are relevant due to the almost aligned configurations Cu-N-C(2), Cu-N(2)-C(2) and Cu-C-C(2).[2]

In complex **10**, R is a hydrogen atom, which is expected to provide a negligible EXAFS signal. On the contrary, the carbon atoms of CH_3 groups of complex **16** are expected to provide a sizable signal.

The distances and mean square relative displacement (MSRD) σ^2 obtained from the refinement are reported in Table 2 of the manuscript for SS contributions. In complex

10 the distances demonstrate a good agreement with the expected molecular structure: the Cu-N nearest neighbour shell is found at 1.96 Å, the Cl is at 2.23 Å from Cu. The Cu-C and Cu-N(2) shells are indistinguishable in complexes **10** and **15** and a single contribution (N = 4) was used (the very similar backscattering amplitude and phase functions of C and N make them difficult to distinguish in the analysis). The analysis of complex **16** requires an additional Cu-C^R contribution at around 1.89 Å. This finding definitively demonstrates the modification of Cu coordination geometry from roughly square planar (complex **10**) to roughly octahedral (complex **16**). This contribution is mandatory to achieve reliable fit; without it the Cu-Cl shell results unphysically short. It is possible that such additional contact originates from the CH_3 residue.

In complex **15** the Cu-N distance is slightly longer, due to the lower Cu(I) electronegativity providing a more loosely bound structure; a single contribution is used to reproduce the Cu-C and Cu-N(2) shells around 2.9 Å, but an additional contribution at around 3.3 Å is required. We likely assume that this last comes out from the C atoms of the phenyl rings linked to P (triphenylphosphine). In order to check the Cu(I) coordination number we refined the EXAFS signal using 2N+2P, 1N+2P and 2N+1P as Cu(I) nearest neighbour shells configurations. The best fit agreements were quantified looking at the weighted residual square function R_W^2 resulting the lowest ($R_W^2 = 0.080$) for 2N+2P with respect to the 1N+2P ($R_W^2 = 0.084$) and 2N+1P ($R_W^2 = 0.089$) models.

Table S1. XPS data analysis results: BE, FWHM, atomic percents and proposed assignments.						
Sample	Signal	BE (eV)	FWHM (eV)	Experimental Atomic Ratios	Theoretical Atomic Ratios	Assignment
4	C1s	284.70<br>286.79<br>288.06<br>289.40<br>291.90	1.47<br>1.47<br>1.47<br>1.47<br>1.47	9.5<br>7.1<br>4.8<br>-<br>1	7.5<br>3<br>2.5<br>-<br>1	C-C aromatic<br>C-N<br>NHCO, C=N<br>COOH impurities<br>C-Cl
	O1s	532.44	2.73	1	1	C=O
	N1s	399.21<br>401.11	2.89<br>2.89	1.6<br>1	1.6<br>1	amine- or amide-like N-C<br>imine-like N=C
	Cl2p	200.44	2.77	1	1	C-Cl
10	C1s	284.70<br>286.35<br>288.12<br>289.63<br>291.24	1.55<br>1.55<br>1.55<br>1.55<br>1.55	10<br>3<br>2<br>-<br>1	5.5<br>3<br>2.5<br>-<br>1	C-C aromatic and alifatic<br>C-N<br>NHCO, C=N<br>COOH impurities<br>C-Cl
	O1s	531.88<br>533.27	1.80<br>1.80			C=O<br>C-O impurities
	N1s	399.70	3.49	1	1	amine- or amide-like N-C
	Cl2p	198.00<br>200.09	1.75<br>1.75	1.4<br>1	1<br>1	Cl-Cu (Cu(II) complex)<br>C-Cl
	Cu2p	936.62	3.24	1		Cu(II)-complex
15	C1s	284.70<br>286.03	1.75<br>1.75	21<br>6	22.5<br>7.5	C-C aromatic and alifatic<br>C-N, C-P

		288.01<br>291.74	1.75<br>1.75	1.8<br>1	1.5<br>1	NCO, C=N<br>C-Cl
	N1s	399.97	2.74	1	1	amine- or amide-like N-C
	O1s	531.53	3.57	1	1	C=O
	$P2p_{3/2}$	131.15<br>138.00	2.00<br>2.00	1.6<br>1	2<br>1	PPh_3<br>PF_6
	$Cl2p_{3/2}$	200.24	1.58	1	1	C-Cl
	$Cu2p_{3/2}$	932.37	2.22	1	1	Cu(I) complex
	F1s	685.88	2.23	F/P = 5.4	F/P = 6	PF_6
16	C1s	284.70<br>286.39<br>288.33<br>289.69<br>291.15	1.62<br>1.62<br>1.62<br>1.62<br>1.62	9.5<br>2.5<br>1.3<br>-<br>1	7.5<br>4.5<br>1.5<br>-<br>1	C-C aromatic<br>C-N<br>NHCO, C=N<br>COOH impurities<br>C-Cl
	O1s	531.72<br>532.98	1.91<br>1.91			C=O<br>C-O impurities
	N1s	400.60	3.46	1	1	amide- or amine-like N-C
	Cl2p	198.01<br>200.13	1.62<br>1.62	1.3<br>1	1<br>1	Cl-Cu (Cu(II) complex)<br>C-Cl
	Cu2p	935.92	2.54	1		Cu(II) complex

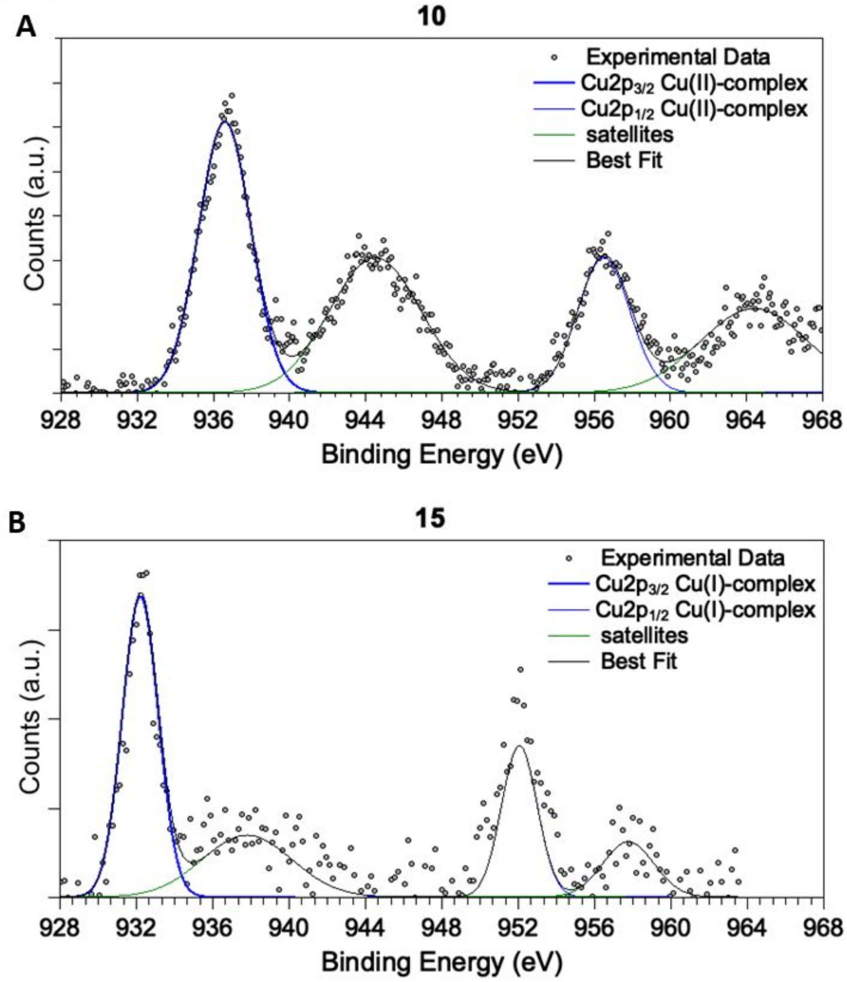

Figure S1. XPS Cu2p spectrum of Cu(II) complex **10** (A) and Cu(I) complex **15** (B).

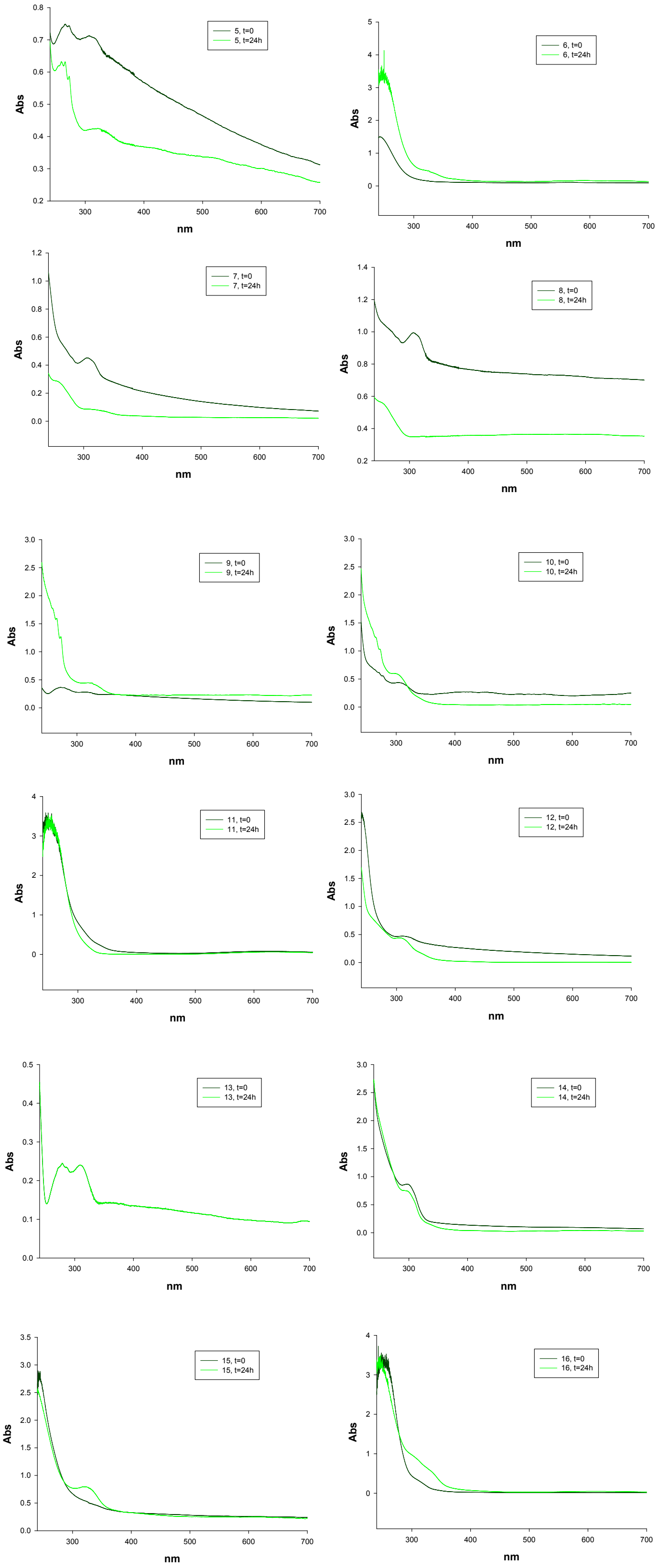

Figure S2. Stability studies. All complexes were dissolved at 50 µM in 0.5% DMSO/RPMI. UV-visible spectra were recorded at t = 0 min and t = 1440 min = 24 h.

References

1. Meneghini, C.; Bardelli, F.; Mobilio, S. ESTRA-FitEXA: A software package for EXAFS data analysis. *Nucl. Instrum. Methods Phys. Res., Sect. B* **2012,** 285, 153-157, DOI: https://doi.org/10.1016/j.nimb.2012.05.027.
2. Meneghini, C.; Morante, S. The active site structure of tetanus neurotoxin resolved by multiple scattering analysis in X-ray absorption spectroscopy. *Biophys. J.* **1998,** 75 (4), 1953-1963, DOI: 10.1016/s0006-3495(98)77636-2.
